# Neurotoxicity of mancozeb-based commercial fungicide in human neuroblastoma SH-SY5Y cells

**DOI:** 10.17179/excli2025-9090

**Published:** 2026-01-08

**Authors:** Evelin G. Cuadros-Buenaventura, Lenin Ramírez-Cando, Ronny A. Ordoñez Sánchez, Johnny Chimborazo, Santiago J. Ballaz

**Affiliations:** 1 School of Biological Sciences and Engineering, Universidad Yachay Tech, Urcuquí, Ecuador; 2 School of Physical Sciences and Nanotechnology, Universidad Yachay Tech, Urcuquí, Ecuador; 3 School of Medicine, Universidad Espíritu Santo, Samborondón, Ecuador

**Keywords:** atomic force microscopy, cytotoxicity tests, mancozeb, neurons, oxidative stress, zinc

## Abstract

Mancozeb, a polymeric dithiocarbamate complex fungicide with zinc and manganese salts, has the potential to be neurotoxic to humans. Unfortunately, the parent molecule maneb has attracted far too much attention, limiting the available evidence on mancozeb neurotoxicity to preclinical research and non-human cells. We sought to evaluate mancozeb cytotoxicity in neuroblastoma SH-SY5Y cells at lower concentrations than those used for maneb in *in vitro* investigations in order to quantify its risk for humans. Commercial mancozeb showed concentration- and time-dependent neurotoxicity in the 3-(4,5-dimethylthiazol-2-yl)-2,5-diphenyltetrazolium bromide reduction test (EC_50_= 5.9 µM and 1.7 µM at 24 h and 72 h respectively). Using the trypan blue exclusion dye, cell death toll reached around 100% after 24- and 72-hour exposure to mancozeb 1 µM and 0.5 µM respectively. Reactive oxygen species generated by mancozeb, which peaked at 4 µM, could be the cause of cell death. The number and length of neurites were concentration-dependently reduced by mancozeb at sub-µM concentrations, and this was accompanied by changes in cell biomechanical characteristics (stiffness) as determined by atomic force microscopy. The uncertainty factor obtained from our cytotoxic studies, when performing risk assessment of mancozeb, varied from 200 to 2000, which may result in detectable neurotoxicity in humans in accordance with international regulatory agencies recommendations.

See also the graphical abstract(Fig. 1).

## Introduction

Mancozeb (MCZ), chemically defined as manganese, zinc ethylene bis-dithiocarbamate (Mn/Zn-EBDC), is a polymeric complex with zinc salt (9 Mn(2+): 1 Zn(2+)); of the group of the dithiocarbamate pesticides, specifically used as a non-systemic (surface acting) fungicide (ECHA, 2024[21]). It is a broad-spectrum, low cost, and highly demanded agrochemical widely used in co-formulations with other fungicides as an anti-resistance treatment given its multiple-site activity mechanism of action. The use of MCZ in agriculture has been controversial due to the putative adverse effects it may have on human health (Dall'Agnol et al., 2021[14]; Cesio and Heinzen, 2024[7]). Despite being banned in the European Union, MCZ is nevertheless one of the most often used fungicides in banana crops for chemical control of the devastating Black Sigatoka disease in tropical countries like Ecuador.

An extensive number of *in vitro*, animal, occupational, and epidemiological studies have raised concerns regarding the safety of MCZ for humans (Tsang and Trombetta, 2007[67]; Rashid et al., 2010[58]; Hoffman et al., 2016[30]; Runkle et al., 2017[60]; Dall'Agnol et al., 2021[14]; Zhang et al., 2023[70]), being the thyroid gland (Skalny et al., 2021[63]) and the reproductive system (Bianchi et al., 2020[5]) the main targets of its harmful effects (Cesio and Heinzen, 2024[7]). The European Chemicals Agency (ECHA) classed MCZ as a reproductive toxin, while the European Food Safety Authority (EFSA) designated it as an endocrine disruptor. Structural similarities between MCZ and maneb (MB or Mn-EBDC), a fungicide that mimics the etiopathogenesis of Parkinson's disease or PD (Meco et al., 1994[45]; Uversky, 2004[68]), add developmental neurotoxicity problems (Mora et al., 2018[47]; Juntarawijit et al., 2020[32]; Fuhrimann et al., 2021[24]) to the increasing list of adverse effects of MCZ exposure. The potential neurotoxicity of MCZ (Domico et al., 2006[18]; Anderson et al., 2021[3]) may not solely rely on its degradation compound ethylenethiourea, the major toxic of concern (Hwang et al., 2003[31]; Cesio and Heinzen, 2024[7]), but it may be blamed on its metallic part (Hoffman et al., 2016[30]; Hernández et al., 2020[28], 2021[29]). 

The evidence of neurotoxic effects of MCZ is scattered in time. Acute (30 min) and chronic (from 24 hours to 15 days) MCZ exposure through food (from 0.2-10 mg/mL in small organisms to 5-10 mg/L in bigger organisms) results in dopaminergic neuron damage in *Caenorhabditis elegans *(Negga et al., 2011[51]; Harrison Brody et al., 2013[27]), *Drosophila melanogaster *(Acosta Saraiva et al., 2021[1]), as well as in oxidative stress in the brain of *Cyprinus carpio* (carp) (Costa-Silva et al., 2018[12]) and *Danio rerio* (zebrafish) (Paganotto Leandro et al., 2021[52]). Neuronal alterations have also been investigated in primary cultures of mesencephalic neurons from rat (Domico et al., 2006[18]), in primary cultures of cerebellar cortex neurons from mice (Peña-Contreras et al., 2016[54]), and in the hypothalamus of mice *in vivo* (Morales-Ovalles et al., 2018[48]). *In vitro* research on MCZ neurotoxicity is not as extensive as MB. Only MB has been challenged in the human neuroblastoma SH-SY5Y cell line (Liu et al., 2022[39]), the gold standard *in vitro* model of dopaminergic neurons in PD (Xie et al., 2010[70]). There is little evidence of MCZ's *in vitro* neurotoxicity in SH-SY5Y cells, which has not been reported in any scientific journals yet but in a thesis of da Silva (2023[13]). Consequently, it is still unclear whether MCZ increases a person´s risk of PD or affects dopaminergic neurons in the same way as MB (Meco et al., 1994[45]; Barlow et al., 2005[4]). Because the neurotoxic risk of MCZ on humans has yet to be documented in scientific literature, the goal of this study was to assess it at critical, low concentrations using the human neuroblastoma-derived SH-SY5Y cell line (Kovalevich et al., 2021[33]). This dopaminergic cell line shows an excellent *in vitro *sensitivity to the potential neurotoxic effects of organic pollutants like pesticides (Lopez-Suarez et al., 2022[41]). 

## Materials and Methods

### Reagents and chemicals

For cell culture, Gibco (Carlsbad, CA) supplied Dulbecco's modified Eagle minimum essential/Ham's F-12 (DMEM/F12) plus Glutamax media fetal bovine serum (FBS), penicillin G/streptomycin mix, and enzyme-free PBS-based cell dissociation buffer. Sigma-Aldrich (St. Louis, MO) provided the 3-[4,5-dimethylthiazol-2-yl]-2,5-diphenyltetrazolium bromide (MTT) reagent and the trypan blue stain. We used commercial wettable powder (WP) mancozeb 80% (Manzate® 80, UPL Ltd., Mumbai, India).

### SH-SY5Y cell culture

The human neuroblastoma cell line SH-SY5Y was a generous donation of Dr. Javier Saez-Castresana (University of Navarra, Spain). Undifferentiated SH-SY5Y cells (immature state) were cultured to confluence (> 80 %) in growth medium [DMEM/F12 (1:1) medium GlutaMAX, 10% v/v fetal bovine serum (FBS), 100 U/mL penicillin G, and 100 µg/mL streptomycin, and kept at 37 °C in a humidified atmosphere of 95 % oxygen and 5 % carbon dioxide. The medium was replaced every 2 to 3 days. After the culture had grown, the cells were harvested using an enzyme-free phosphate-buffered saline (PBS)-based cell dissociation solution. 

We chose undifferentiated cells since retinoic acid (RA) differentiation (Cheung et al., 2009[8]) significantly changes SH-SY5Y cells frequently making them more resilient and resistant to Parkinsonian specific neurotoxins like 6-OHDA (6-hydroxydopamine) or overall oxidative stress because of improved defensive systems (Schneider et al., 2011[62]). Although not exactly resembling mature neurons, undifferentiated SH-SY5Y cells have a star-like shape with short neurites that provide a useful expandable model for general *in vitro* neurotoxicity research. The use of RA differentiated cells is more relevant when examining the neurobiology of Alzheimer's diseases (de Medeiros et al., 2019[15]).

### MCZ treatment

The concentrations of sheer fungicide were set up according to the percentage of the active ingredient specified by the manufacturer (80 % w/v). Mancozeb or MCZ (zinc;manganese(2+);*N*-[2-(sulfidocarbothioylamino)ethyl]carbamodithioate; chemical formula: C_40_H_60_Mn_9_N_20_S_40_Zn (M.W.= 2664 g/mol); CAS Ref.#: 8018-01-7) were freshly prepared in a stock solution of sterile water just before treating the cells and then serial dilutions were directly made in the growth medium (covered concentration range: 0.1 to 16 µM). Because of the poor solubility of MCZ in water, Manzate® contains the active ingredient in a wettable powder formulation. Thanks to the combination of wetting excipients, the fungicide quickly and evenly disperses when added to water. As a result, the serial dilutions of MCZ were entirely clear, even though the stock solution was slightly cloudy.

### Neuronal-viability evaluation

The first measure of cell viability was the reduction of the thiazolyl blue tetrazolium bromide, or MTT dye, to formazan (Mosmann, 1983[50]). In 96-well microplates, cells were seeded at a density of 6 x 10^4^ cells per well (200 µL) for MTT assays. After a 48-hour incubation period at 37 ºC, a stock of MTT salt (5 mg/mL in PBS) was diluted tenfold in the culture medium. The most active mitochondrial reductase enzymes of living cells converted the yellow MTT salt to purple formazan after a 2-hour incubation at 37 °C, enough time for formazan crystals to form in SH-SY5Y cells (Ramirez-Cando et al., 2024[57]). After aspirating the medium, the formazan crystals were solved in 100 µL of pure DMSO. Using a microplate reader (Rayto RT-2100C Microplate Reader, Rayto Life and Analytical Sciences Co. Ltd., China), the optical density (O.D.) was measured at 560 nm with the appropriate DMSO serving as a blank. The absorbance of the control (non-treated) wells was set to 100 to standardize the absorbance values of the treated wells. A well was treated with 10 % DMSO to serve as a positive cell death control. Eight duplicates of each treatment condition were conducted for every independent experiment.

Further information on cell viability was obtained by measuring the percentage of viable cells that remained intact after applying the vital-dye trypan blue (TB) exclusion test (Strober, 2015[65]). Cells were seeded at a density of 5 x 10^3^ cells per well (400 µL) in 24-well plates and MCZ treatments (0, 0.1, 0.25, 0.5, 1, and 2 µM) were applied 48 hours later. Analysis of cell viability with exclusion dye was done 24 and 72 hours after treatment. Under the light of an inverted microscope (Motic AE31E, Motic China Group Co., Hong Kong, China), TB was dissolved directly into the culture medium (0.02 % v/v) to assess the fraction of dead (TB-tangible) cells versus total cell counts at 200-fold magnification. Pictures were taken with a digital camera connected to MotiConnect software (Motic China Group Co., Hong Kong, China). Cell counts were performed using the free ImageJ software (http://imagej.nih.gov/ij) on a minimum of 500 cells in several fields (ten replicates per treatment condition). 

### Neurite outgrowth

Endpoints for screening neurotoxic effects also included the impairment of important developmental processes such as neurite outgrowth inhibition (Lee et al., 2022[37]). Up to 400 µL of culture medium was used to seed cells at a density of 5 x 10^3^ cells/well in 24-well plates (4 wells per concentration). After 48 hours, MCZ (0, 0.1, 0.2, 0.5, 1 and 2 µM) was administered and the 24 and 72-hour treatment´s visual appearance of the cells was examined in phase-contrast images on ImageJ. Each well was the subject of digital micrographs that were saved on a PC. Neurite-like structures or neurites were measured and examined in ten replicates, with at least 500 cells in each treatment condition. The number of cells displaying neurites that were twice as long as the width of the cell body was used to measure neurite outgrowth. To rule out artifacts from varying cell counts, the averaged neurite length per cell was assessed in relation to control (untreated or 0µM) (Lee et al., 2022[37]).

### Evaluation of ROS generation

Reactive oxygen species (ROS) were measured in treated cells using the probe 2′,7′-dichlorodihydrofluorescein diacetate (H_2_DCF-DA), which is an indirect and non-specific biomarker of oxidative stress (Wang et al., 1999[69]), as our laboratory previously described (Ramirez-Cando et al., 2023[56]; 2024[57]). In brief, cells were seeded onto coverslips placed in Petri dishes at a density of 6 x 10^6 ^cells/ Petri dish with 8 mL of media. These cells were then allowed to proliferate for 48 hours, and finally the fungicide was applied for a 24-hour period. Following treatment, the cells were incubated in a growth medium with 10µM of DCF-DA for 30 minutes. They were then fixed for 10 minutes in ice-cold PBS containing 3.7 % formaldehyde, rinsed three times with PBS, allowed to dry on air, and mounted on glass slides using Entellan^TM^ rapid mounting medium (Merck-Millipore, Germany). Cells were photographed in six fields per experimental condition at a magnification of 200x with the assistance of a Leica DM4000 B imaging fluorescence microscope (Leica Microsystems CMS GmbH, Wetzlar, Germany). Utilizing excitation and emission filters set at 485 ± 10 nm and 530 ± 12.5 nm, respectively, fluorescence intensity was calculated with the use of a blue filter (450-490 nm) and the Leica Application Suite X software for research. Finally, ImageJ was used to evaluate the microphotographs (four images per treatment condition).

### Atomic Force Microscope (AFM) sample preparation and analysis

The corresponding calculations were performed following the cell counting to achieve an initial cell density of 2.5 x 10^4^ cells/mL. Cells were cultured on coverslips (22 x 22 mm with a thickness of 0.13-0.17 mm) placed in four glass Petri dishes (ϕ = 30 mm). The Petri dishes were allocated into one control Petri dish and three Petri dishes for different MCZ concentrations. After placing the coverslips inside the Petri dishes, 25 mL of the DMEM solution with cells was poured into each Petri dish. The Petri dishes were incubated at 37 °C in a humidified O_2_ (95 %) and CO_2_ (5 %) atmosphere for 24 hours. Cells were then treated with MCZ at 0.1, 0.25, and 1.0 µM for 24 and 48 hours, since pilot studies demonstrated that this concentration range worked out better for morphological analysis. Samples were incubated in 3.7 % formaldehyde/PBS solution at room temperature for 20 minutes to fix cells and subsequently washed in distilled water for 3 seconds.

The Atomic Force Microscope (AFM, NX7 model, Park System) was equipped with an integrated On-axis Optic featuring a Vision Camera and an objective lens with 10x magnification for biomechanical analysis. An NSC36 Type A cantilever (Park System, Suwon, Korea) was utilized for contact analysis according to manufacturer's recommendation for contact force exceeding 0.2N/m. The AFM setting was calibrated following the manufacturer's specifications to ensure accurate analysis of the samples. Data from the AFM study of the cell's mechanical properties were collected using the XEI software (TEST Park Systems Corp, Suwon, Korea). Among the several AFM measurements, the sample analysis focused primarily on Young's modulus.

### Statistics

The Rstudio free software (https://posit.co/download/rstudio-desktop/) was used for statistical analysis (Supplementary material includes R codes). To measure neurite outgrowth and % of dead cells (vita dye exclusion test) replicates were conducted in four wells per MCZ concentration, results were expressed as median ± IQR to provide a clearer view of data distribution and variability capturing more information (based on boxplot), and later analyzed through the one-way ANOVA combined with the Dunnet´s test as the *post-hoc* analysis.

In the case of MTT data, data were normalized with respect to control, and a scatter graph was plotted from which a concentration-response curve was adjusted by a four-parameter log-logistic function utilizing the 'drm()' function within the RStudio software package 'drc()' for concentration-response analysis. The logistic function applied for this analysis is given by the expression:


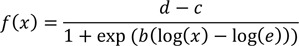


where ***b*** represents the relative slope around ***e***, ***c*** the lower limit, ***d ***the upper limit, and ***e ***the EC_50_.

The relative intensity of ROS was plotted on a scatter graph. Data underwent normalization against control values. A 4^th^ order polynomial concentration-response curve was selected as the best fit as follows:


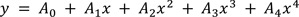


Lastly, using Monte Carlo simulations, an ANOVA was performed on the biomechanical characteristics of the cells acquired from a single AFM experiment and subsequent repeated results (up to 500 per treatment condition). Dunnett's test was performed as the *post-hoc* test. *P* < 0.05 was regarded as statistically significant. for all data analysis.

### Ethics

Ethical approval was not required to use SH-SY5Y cells because they are a commercially available, established cell line, not primary human cells taken directly from a patient. In other words, no human subjects were involved, since SH-SY5Y cells are considered a pre-existing source for research.

## Results

### Viability of SH-SY5Y cells after MCZ treatment

**Figure 2:**
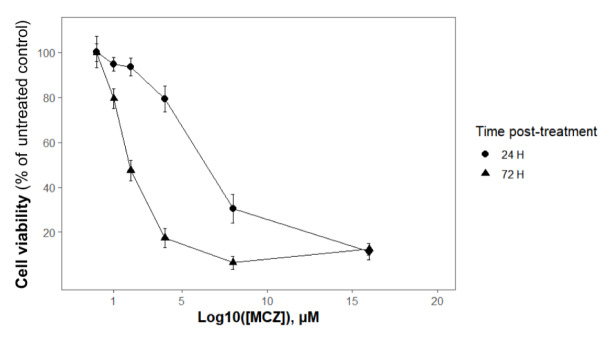
Toxicity of MCZ in SH-SY5Y neurons according to the MTT test. Curves represent cell viability (% of untreated control) after 24- and 72-hour exposure to increasing concentrations (from 1 µM to 16 µM) of MCZ. Concentration-response curves were created using the Logistic Sigmoidal function type 1 (see the Statistics section) based on the parameter's values listed in Table 1*. *Values derived from seven independent experiments (eight replicates per experiment)

The interest was to know whether acute (24 hours) to sub-chronic (72 hours) exposure to increasing concentrations (0.5 to 16 µM) of MCZ fungicide altered cell viability. Figure 2(Fig. 2) represents the concentration-response curves, plotted as the percentage of the untreated control, of the MTT viability test at different treatment periods. The ANOVAs of the MTT test results revealed a significant treatment effect at 24 hours (*F*(5,42) = 53.90, *P *< 0.001) and 72 hours (*F*(5,42) = 100.30, *P *< 0.001)). The adjusted R-squared values were set above 0.7 in both cases, which indicated a good correlation between the model predictions and real values.

**Figure 3:**
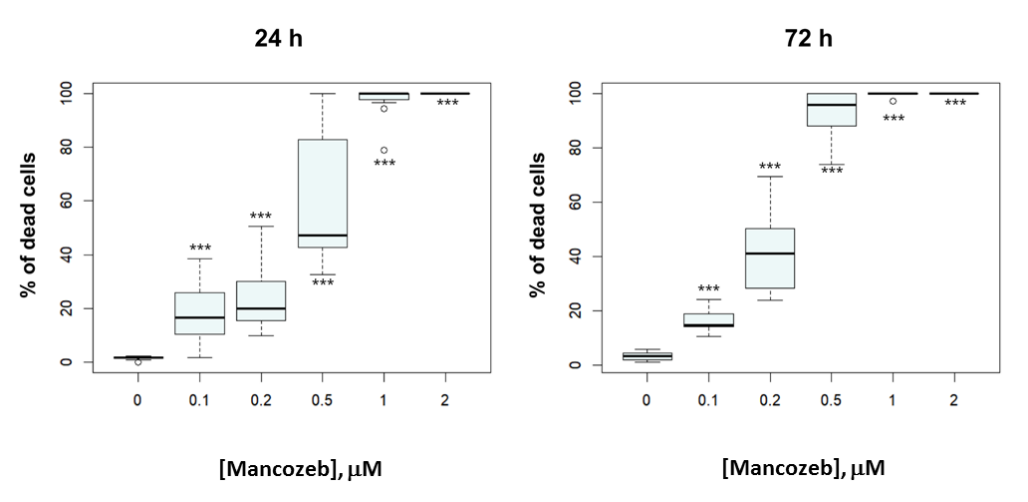
TB test-based MCZ toxicity. Box plots indicate the percentage of dead cells after 24- and 72-hour treatments. Values were expressed as median and interquartile distance for ten replicates to more accurately depict the ANOVA results. ***P < 0.001 compared to untreated controls

**Figure 4:**
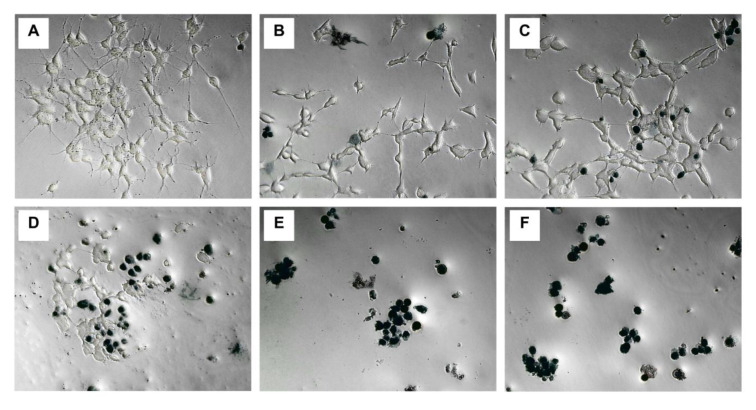
Representative phase contrast micrographs of SH-SY5Y neurons after 24-hour treatment with MCZ. Notice dead cells stained with TB. Fungicide concentrations: (A) 0 µM, (B) 0.1 µM, (C) 0.25 µM, (D) 0.5 µM, (E) 1 µM, and (F) 2 µM

Because the MTT assay is no longer acceptable as a single measure of cell viability, other parameters were required to determine whether MCZ was truly causing cell death. To corroborate MTT data, living cell cultures were examined for the presence of TB-tangible (dead) cells (Figures 3(Fig. 3) and 4(Fig. 4)). The ratio of dead cells caused by the fungicide concentration-dependently increased after 24 (*F*(5,60) = 117.2, *P *< 0.001) and 72 hours (*F*(5,60) = 357.1, *P *< 0.001) of treatment. AMCZ concentrations over 1µM and 0.5µM resulted in cell death rates of almost 100% following 24- and 72-hour exposure.

### MCZ effects on neurite outgrowth and cell morphology

**Figure 5:**
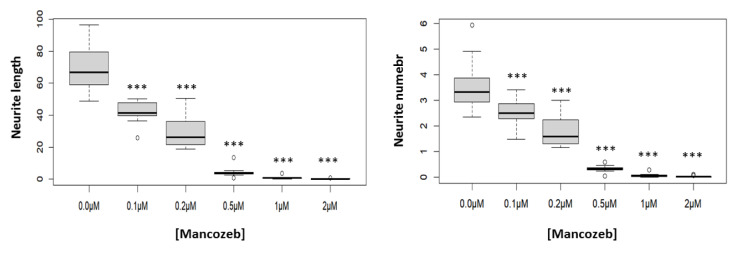
Effects of 24-hour treatment with MCZ (0.1-2 µM) on neurite outgrowth and branching. The graphic displays the medians and interquartile distances for ten replicates to more accurately depict the ANOVA results. ***P < 0.001 compared to untreated controls

Figure 5(Fig. 5) shows the concentration-dependent evolution of neurite outgrowth after 24-hour exposure to MCZ. As shown in Figure 5(Fig. 5), there was a significant treatment effect in the average neurite length per cell (*F*(5,60) = 120.4, *P *< 0 .001); as well as in the number of neurites per cell (*F*(5,60) = 81.9, *P *< 0.001). The characteristic polygonal-shaped soma of SH-SY5Y cells gradually transformed into a more circular shape, while concentration-dependently decreasing in the number and length of neurites per cell.

### MCZ-induced ROS

**Figure 6:**
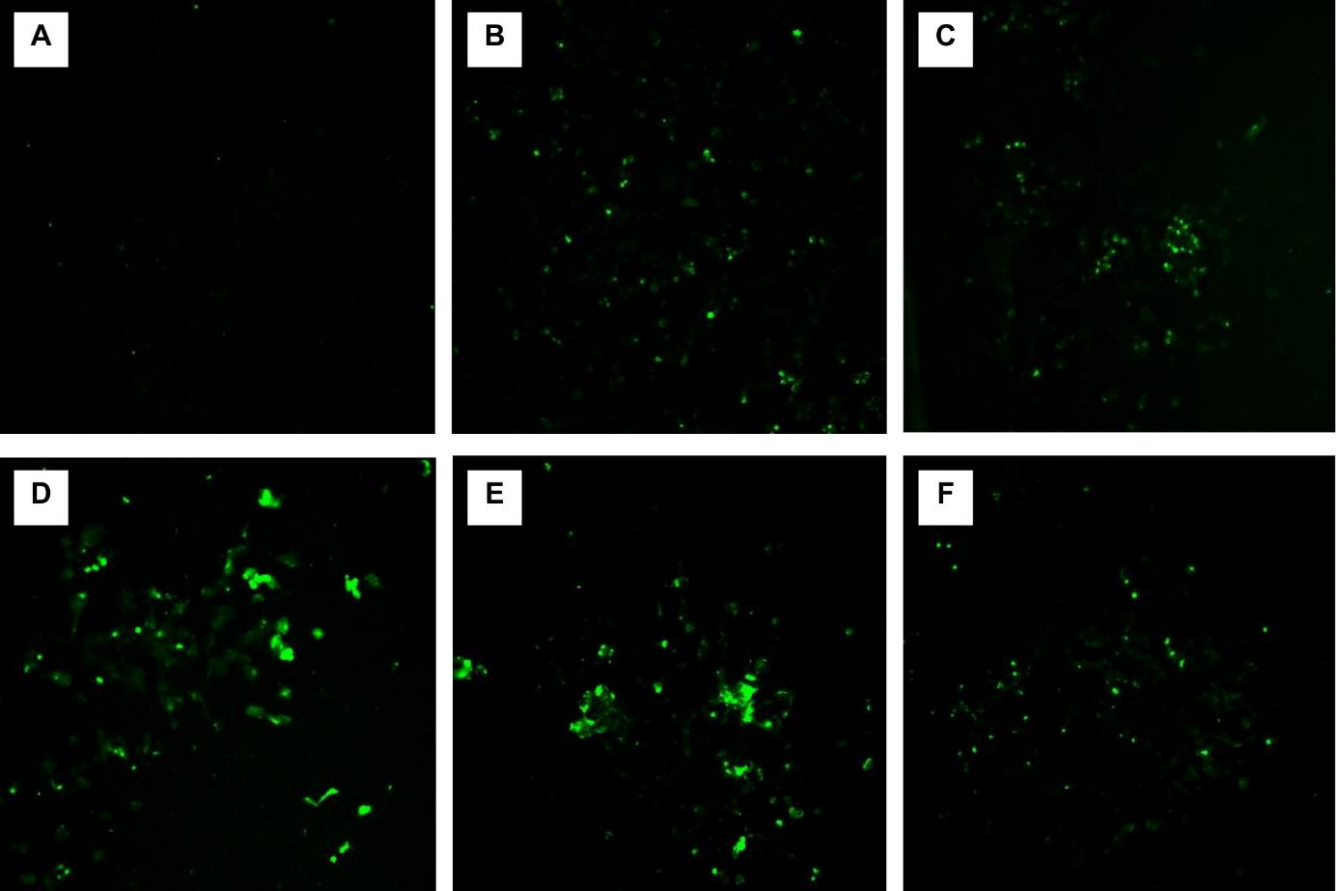
Representative micrographs of SH-SY5Y cells after 24-hour MCZ treatment under fluorescence microscopy. Images (20x magnification) were obtained using the green channel (excitation and emission wavelengths: 485 nm and 530 nm respectively). [MCZ]: (A) untreated control, (B) 1 µM, (C) 2 µM (D), 4 µM, (E) 6 µM, and (F) 10 µM

**Figure 7:**
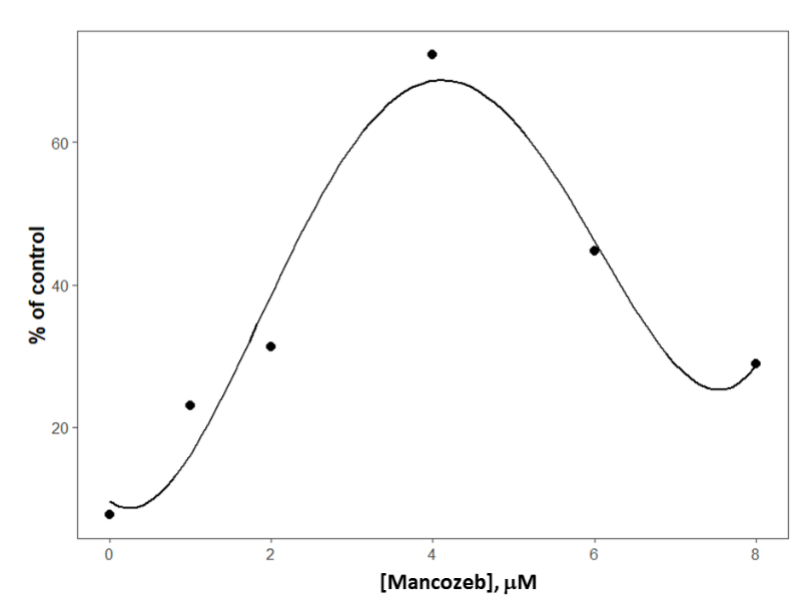
Scatter plot of concentration-response of fluorescence emitted by SH-SY5Y cells after 24-hour treatment with MCZ. The concentration-response curve was adjusted to a 4^th^ grade polynomial response curve based on the parameters contained in Table 2.

The DCHF-DA reagent exhibited a noticeable increase in fluorescence in ROS generating cells (Figure 6(Fig. 6)). The results obtained after 24-hour treatment with MCZ suggested that the fungicide prompted a significant increase in ROS levels compared to control (as no treatment). ROS level reached a maximum at 4 µM of MCZ to posteriorly decrease due to the massive cell death at higher concentrations. The concentration-response curve of ROS generation by MCZ treatment in SH-SY5Y cells moderately followed a biphasic model given by a 4^th^-grade polynomial curve in a range from 1 to 10 µM (Figure 7(Fig. 7)). The adjusted R-squared value was set to 0.754 (*F*(5,24) = 9.89, *P* = 0.3272).

### Young´s modulus analysis 

**Figure 8:**
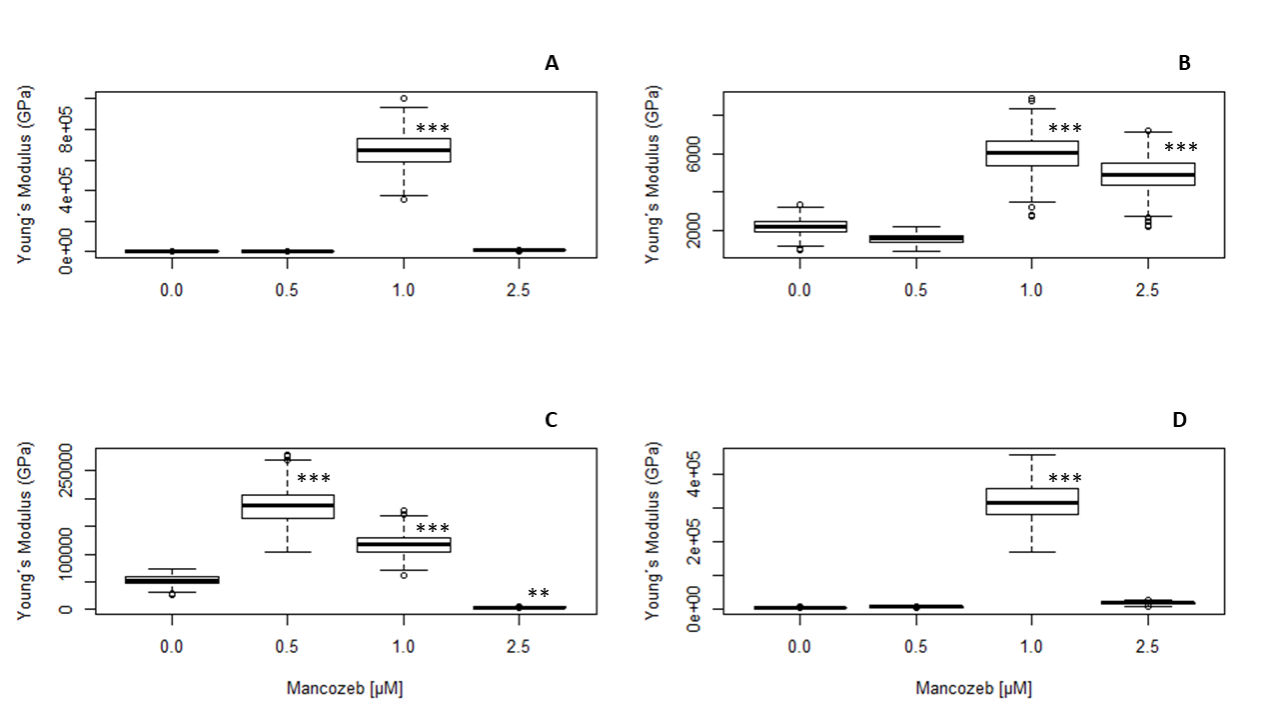
AFM analysis of MCZ (0.1 to 1 µM) on the elastic Young´s modulus. Graphic expressed as median and interquartile distance complementary to one-way ANOVA of one real-life experiment and subsequent fifty Monte Carlo simulations. Data expressed as the percentage of change relative to the modulus´ basal value. (A) Nucleus after 24-hour treatment, (B) axon after 24-hour treatment; (C) nucleus after 48-hour treatment; and (D) axon after 48-hour treatment. ***P < 0.001 compared to the untreated control

**Figure 9:**
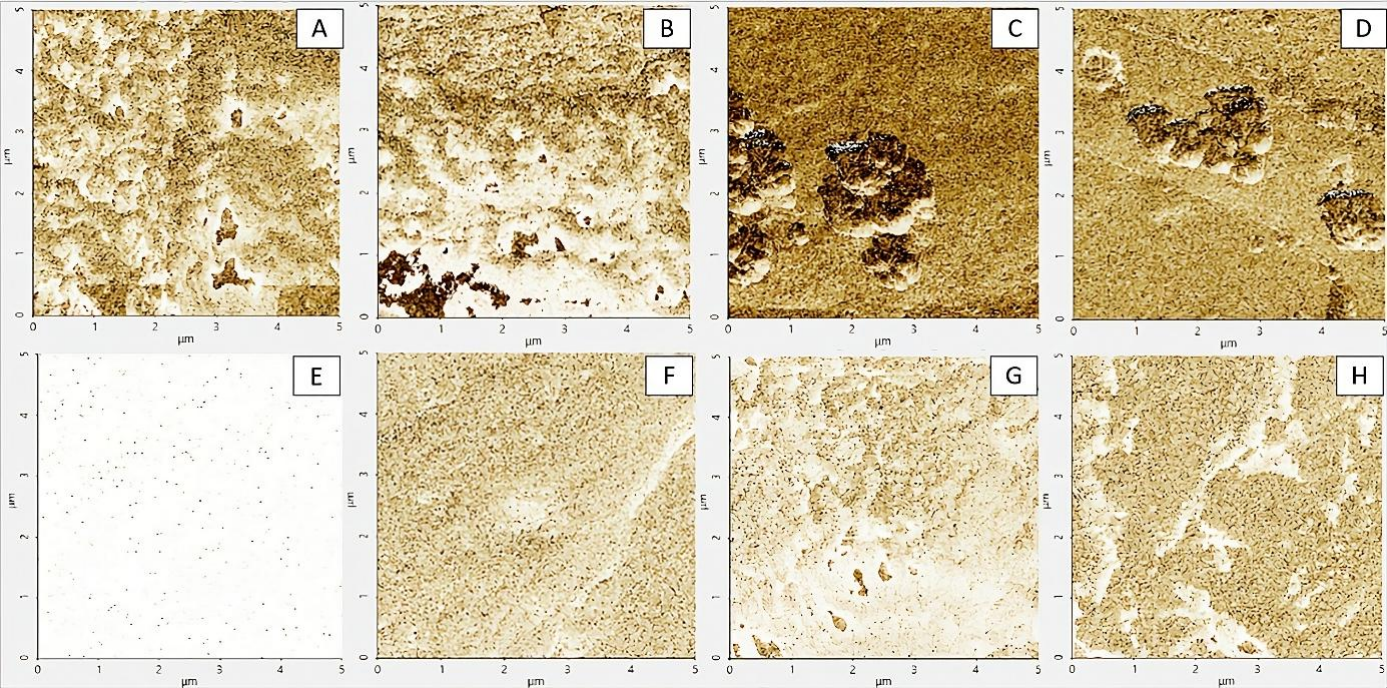
Representative elastic Young´s modulus images of cultured SH-SY5Y cells following 24-hour treatment with MCZ. The following are the 5 x 5 µm AFM images: The nucleus and axon of untreated (control) cells are shown in (A, B); cells treated with MCZ (0.1 µM) are shown in (C, D); cell treated with MCZ (0.25 µM) are shown in (E, F); and cells treated with MCZ (1 µM) are shown in (G, H)

Young's modulus was used to evaluate the biomechanical response of cells, which depends on a combination of intrinsic cellular factors (like cytoskeleton, cellular shape, and molecular motors) and extrinsic factors (such as external forces, matrix stiffness, and biochemical cues), to different concentrations of MCZ. Statistical analysis via ANOVA of 500 Monte Carlo-outcomes mimicking one real-life AFM experiment revealed a significant treatment effect on the modulus of elasticity of both cell nuclei (24-hour MCZ: *F*(3,1996) = 175.15; *P *< 0.001; 48-hour MCZ: *F*(3,1996) = 876.06, *P*< 0.001) and axons (24-hour MCZ: *F*(3,1966) = 477.18; *P *< 0.001; 48-hour MCZ: *F*(3,1996) = 1643.21, *P *< 0.001) (Figure 8(Fig. 8)). Subsequent Dunnett's test pinpointed robust differences across treatments, with statistically significant deviations from untreated (control) cells. As shown in Figure 8(Fig. 8), the increase of Young's modulus was the largest after exposure to 0.25 µM of MCZ in both the axon and the nucleus, regardless of treatment time. This phenomenon suggests a possible activation of cellular response mechanisms that increased stiffness. This concentration-dependent behavior suggests that, whereas low MCZ concentrations fortify the cellular structure, higher ones undermine it (see Figure 9(Fig. 9)). More interestingly, the pattern of elastic modulus changes in the nucleus after 24-hour MCZ treatment (Figure 8A(Fig. 8)) was like the axonal response to 48-hour MCZ treatment (Figure 8C(Fig. 8)), thus highlighting nuanced, concentration-dependent effects on cell mechanics shifting from the nucleus to cell body throughout time. After 48-hour treatment, all tested concentrations of MCZ significantly influenced the nucleus's mechanical properties, underscoring the pronounced impact on cellular rigidity (Figure 8C(Fig. 8)), while in axons, only the treatment with 0.25 µM of MCZ produced a robust stiffness increase (Figure 8D(Fig. 8)).

See also Table 1(Tab. 1) and 2(Tab. 2).

## Discussion

The primary results of this research showed that MCZ was cytotoxic in neuroblastoma SH-SY5Y cells, a gold standard *in vitro* model, even at sub-microM concentrations. This range can be utilized to research neurotoxic effects in humans at more realistic levels. In this investigation, we sought to determine the critical range of neurotoxic concentrations of MCZ, which turned out to be smaller than that reported for MB, a closely related dithiocarbamate molecule, in similar *in vitro* toxicological tests (Choong and Say, 2011[9]; Caputi et al., 2015[6]; Anderson et al., 2018[2], 2021[3]; Liu et al., 2022[39], 2023[40]; Conde et al., 2023[11]). Because MB does not contain Zn(2+) in its molecular structure, our findings could suggest that Zn(2+) and Mn(2+) together may have an impact on the MCZ´s higher neurotoxicity compared to MB.

To stimulate a more realistic exposure scenario, we used commercially available MCZ at concentrations hundreds of times lower than the levels of environmental exposure to the fungicide (Lori et al., 2021[42]) based on the refining of uncertainty/safety factors (Dorne and Renwick, 2005[19]). Following a 24-hour treatment, the EC_50_ for MCZ in our investigations ranged from 0.5 µM (trypan blue test) to 6µM (MTT test), a concentration lower than the reported EC_50_ for sheer MCZ (10 µM) compound in functional viability experiments utilizing mesencephalic-striatal primary co-cultures from rat brain (Soleo et al., 1996[64]). Additionally, some evidence suggests that the generation of ROS may contribute to the decline in the functional viability of rat mesencephalic neurons following a 24-hour exposure to 30 µM of MCZ (Domico et al., 2007[17]). In our experiments, ROS generation happened at a far smaller range of concentrations (1 to 10 µM) after 24-hour treatment with MCZ. As per the principle of “Use of a LOAEL rather than a NOAEL as the critical effect” (Maier, 2005[43]) most researchers currently utilize an average range of sheer MCZ concentrations higher than we used in our study. For the first time, we described the concentration-dependent effects of sub-µM concentrations of MCZ on neurite outgrowth and cell morphology in SH-SY5Y cells. We also made the decision to use Young's modulus as a measure of material elasticity (Kuznetsova et al., 2007[36]), because of the potential of MCZ to alter cellular structure and behavior. Because a higher risk of developing a neurodegenerative disease (Fang et al., 2014[23]) is linked to increased cell surface roughness and stiffness (Meyer and Amer, 1990[46]), MCZ's concentration-dependent effects in cell rigidity indicated its importance in preserving cell function and survival. All the points suggest that the use of non-human *in vitro* models for dopaminergic neurons may have underestimated the neurotoxic potential of MCZ in inducing mostly neural degeneration (Negga et al., 2011[51]; Harrison Brody et al., 2013[27]; Peña-Contreras et al., 2016[54]).

Due to their similar chemical structures, MB has dominated *in vitro* neurotoxicity research tying it to Parkinson's disease and neurodegeneration (Liu et al., 2022[39]). In contrast to most of the cell *in vitro* research on MB (Choong and Say, 2011[9]; Caputi et al., 2015[6]; Anderson et al., 2018[2], 2021[3]; Liu et al., 2022[39], 2023[40]; Conde et al., 2023[11]), we detected concentration-dependent neurotoxic effects for our 24-hour MCZ exposure in SH-SY5Y cells at tree- to ten-fold lower concentrations. More researchers utilize MB concentrations ranging from 10 μM to 50 μM to achieve the neurotoxic effects, except for one study (Roede et al., 2011[59]) that used a concentration range like ours (1-10 µM). While it has been documented that elevated levels of MCZ or MB can cause mitochondrial dysfunctions in SH-SY5Y cells (Domico et al., 2006[18]) and oxidative stress (Domico et al., 2007[17]), the remarkably low EC_50_ values observed in this investigation could indicate additional neurotoxic mechanisms, like the activation of the KCNQ2 channel, a fundamental player modulating neuronal excitability (Li et al., 2013[38]). Chemically speaking, MCZ is a polymeric complex of Mn(2+) and Zn(2+) with the ethylene bis-dithiocarbamate anion ligand. The presence of Zn(2+) at a ratio of 1 to 9 regarding Mn(2+) is what chemically separates MCZ from MB. Manganism is connected to neurodegeneration (Dobson et al., 2004[16]; Hernández et al., 2020[28]), but almost nothing is known about zinc-induced neurotoxic effects (Morris and Levenson, 2017[49]). It is possible that MCZ neurotoxicity results from a complex interaction between the organic anion, and the presence of both cations (Li et al., 2013[38]), since Zn(2+) cytotoxicity *in vitro* strongly depends on the chemical makeup of the anion counterpart (Pavlica et al., 2009[53]).

We focused on Zn(2+) because it could be the reason behind MCZ´s greater than what was documented for MB. In-house experiments (unpublished observations) verified that MCZ significantly increased the conductivity of the solution when dissolved in distilled water due to the breakage of the polymeric complex and subsequent cation release. The cytoskeleton organization may be impacted by the intracellular built-up of Zn(2+) (Kress et al., 1981[34]) and the dithiocarbamate monomer (Schmuck et al., 2002[61]). Furthermore, some evidence points to Zn(2+) disrupting SH-SY5Y cells' bilayer structure of the membrane (Suwalsky et al., 2009[66]) This body of evidence may help to explain the alteration in these cells' mechanical characteristics that MCZ causes, as seen in the AFM investigations.

The essential use of MCZ in a wide variety of agricultural products for daily consumption is directly related to the passive exposure of the general population to this agrochemical and its metabolites (EFSA, 2020[22]). Risk assessment analysis uses uncertainty factors to make up for information gaps about test results accuracy and the difficulty of estimating health impacts (Dorne and Renwick, 2005[19]). An agriculture worker´s skin can absorb a median of 0.9 µg/Kg bw/day (Mandić-Rajčević et al., 2020[44]), which is close to the Acceptable Operator Exposure Level (AOEL) of MCZ (3.5 µg/Kg bw/day; EC, 2009[20]). For this reason, an exposed agriculture worker´s estimated blood content of MCZ may be as high as 0.48 nM (Mandić-Rajčević et al., 2020[44]). Based on the lowest observed adverse effect level (LOAEL) in our experiments (0.1-1 μM), the highest uncertainty factor derived from the ratio of LOAEL to AOEL was between 200 and 2000 (Ramirez-Cando and Ballaz, 2025[55]). For humans, exposure via skin and lungs is acknowledged. A small occupational study measured dermal patches and found correlation between dermal pad ethylethiourea and urinary metabolite (Colosio et al., 2002[10]). This information related to toxicokinetic points out a plausible exposure distribution factor of 0.1 % to contact neural systems (considering uncertainty factor and ADME for MCZ). EPA and EFSA reports dose range 40-1200 mg/kg for genotoxicity and hepatotoxicity (Dall'Agnol et al., 2021[14]; EFSA, 2020[22]). Converting doses tested in this study fall within the reported range with a max of 60 mg/kg for cytotoxicity and 15 mg/kg for ROS as critical biological effect.

We ruled out the interference of formulation-related excipients (<1 µg/mL in culture medium) in the observed neurotoxicity of mancozeb, since regulatory agencies such as FDA and EPSA require excipients used in agrochemicals to be safe to avoid endangering the environment or human health. Furthermore, the product´s information sheet states that the hazardous ethylene thiourea (ETU) metabolite was present in less than 1g/kg (<1 ng/mL in culture medium). One possible limitation of the study is that the two metals (Mn(2+) and Zn(2+)), were not used as soluble (chloride) form in the experiments (Domico et al., 2006[18]). However, Mn(2+) and Zn(2+) are examples of soluble ions that quickly attached to proteins and other molecules (Haase et al., 2015[26]; Krężel and Maret, 2016[35]), altering their behavior in ways that may not accurately represent the neurotoxic effects of MCZ. Additionally, soluble ions can override the tight transporters that cells have for critical metals, resulting in artificial neurotoxic effects.

In addition to some preliminary research, the thesis of da Silva (2023[13]) is the first evidence of mancozeb´s neurotoxicity in a model of human dopaminergic neurons. This eliminates the problem of species extrapolation and offers a more pertinent approach for studying its neurotoxicity risk in humans. The presence of zinc (Zn(+2)) in mancozeb significantly alters its stereochemistry compared to other dithiocarbamates like maneb. This change in stereochemistry, or special arrangement of atoms within the molecule, is believed to contribute to mancozeb´s increased toxic potential. To protect the nervous system from oxidative stress-induced damage, a network of protein filaments is essential for preserving the integrity of the neuronal membrane and overall cell function (Gardiner et al., 2013[25]). It is nevertheless a target for this damage. We hypothesize that the polymer´s oxidative potential may be increased by stereochemistry that zinc cation imparts.

## Conclusions

In conclusion, the detrimental effects on SH-SY5Y cells demonstrate that MCZ may have even greater neurotoxicity than its companion chemical MB for humans. Given that Zn(2+) is what chemically distinguishes MCZ from MB, our findings suggest that the cation may play a role in MCZ neurotoxicity. Following our study of MCZ's risk assessment, more studies using additional cell lines are needed to validate these results and gather information on their neurotoxicity *in vivo*.

## Notes

Lenin Ramírez-Cando and Santiago J. Ballaz (School of Medicine, Universidad Espíritu Santo, Av. Samborondón 5, 0901952-Samborondón, Ecuador; E-mail: sballazg@gmail.com) contributed equally as corresponding author.

## Declaration

### Acknowledgments

We are grateful to the *Programa de las Naciones Unidas para el Desarrollo *(PENUD) of the Ecuadorian Government. The authors specially thank Prof. Lola de Lima and Prof. Carlos Reinoso for their technical support with the chemical characterization of MCZ and with the AFM respectively.

### Funding

This work was supported by the *Secretaria Nacional, Ciencia, Tecnología e Innovación *(SENESCYT) grant of the Ecuadorian Government (Ref. PIC-18-INE-YACHAY-002) to Santiago J. Ballaz and Lenin J. Ramirez-Cando.

### Declaration of competing interest

None.

### Author contribution statement

LJR-C and SJB: Conceived and design the study, analyzed and interpreted the data, and contributed with reagents, materials, analysis tools or data; EGC-B: Performed the *in vitro* experiments, and wrote the original draft; JC and RAO: Performed AFM experiments and analysis. All the authors wrote and approved the final version of the manuscript.

### Data availability

The datasets generated analyzed during the current study are available from the corresponding author on reasonable request.

### Disclosure of delegation to generative AI

The authors declare that generative AI was not used during the writing and the research process. The authors are solely responsible for the finished manuscript.

## Supplementary Material

Supplementary information

## Figures and Tables

**Table 1:**
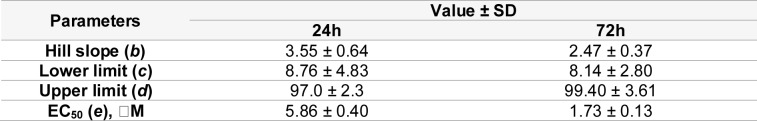
Concise table of parameter values for concentration-response curve modeling based on MTT-based cell viability at 24 and 72 hours

**Table 2:**
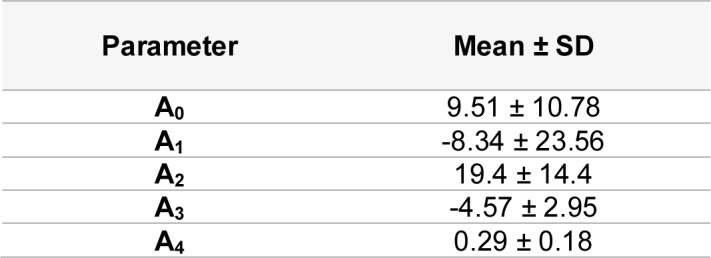
Condensed table of parameter values (derived ROS assessment trials) for modeling the concentration-response curve based on relative intensity. Values derived from 4 replicates

**Figure 1:**
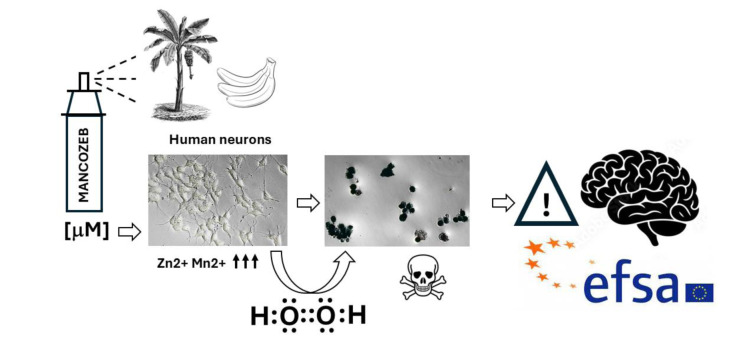
Graphical abstract
