# Supplementary material for: Neurotoxicity of mancozeb-based commercial fungicide in human neuroblastoma SH-SY5Y cells
Source: EXCLI J. 2026 Jan 8;25:133–49. doi: 10.17179/excli2025-9090 (PMC12946439; doi:10.17179/excli2025-9090)
Supplement: Supplementary information [file EXCLI-25-133-s-001.pdf]

EXCLI Journal 2026;25:133-149 – ISSN 1611-2156
Received: November 06, 2025, accepted: December 15, 2026, published: January 08, 2026

Supplementary information to:

Original article:

NEUROTOXICITY OF MANCOZEB-BASED COMMERCIAL FUNGICIDE IN HUMAN NEUROBLASTOMA SH-SY5Y CELLS

Evelin G. Cuadros-Buenaventura[1], Lenin Ramírez-Cando[1*], Ronny A. Ordóñez[1], Johnny Chimborazo[2], Santiago J. Ballaz[3*]

[1] School of Biological Sciences and Engineering, Universidad Yachay Tech, Urcuquí, Ecuador
[2] School of Physical Sciences and Nanotechnology, Universidad Yachay Tech, Urcuquí, Ecuador
[3] School of Medicine, Universidad Espíritu Santo, Samborondón, Ecuador

* **Corresponding authors:** Prof. Santiago J. Ballaz, PhD, School of Medicine, Universidad Espíritu Santo, Av. Samborondón 5, 0901952-Samborondón, Ecuador. E-mail: sballazg@gmail.com
Prof. Lenin J. Ramírez-Cando, PhD, School of Biological Sciences and Engineering, Universidad Yachay Tech, Av. Universitaria S/N, 10102 Urcuqui, Ecuador. E-mail: lramirez@yachaytech.edu.ec

https://dx.doi.org/10.17179/excli2025-9090

This is an Open Access article distributed under the terms of the Creative Commons Attribution License (https://creativecommons.org/licenses/by/4.0/).

ABSTRACT

Mancozeb, a polymeric dithiocarbamate complex fungicide with zinc and manganese salts, has the potential to be neurotoxic to humans. Unfortunately, the parent molecule maneb has attracted far too much attention, limiting the available evidence on mancozeb neurotoxicity to preclinical research and non-human cells. We sought to evaluate mancozeb cytotoxicity in neuroblastoma SH-SY5Y cells at lower concentrations than those used for maneb in *in vitro* investigations in order to quantify its risk for humans. Commercial mancozeb showed concentration- and time-dependent neurotoxicity in the 3-(4,5-dimethylthiazol-2-yl)-2,5-diphenyltetrazolium bromide reduction test (EC_{50}= 5.9 µM and 1.7 µM at 24 h and 72 h respectively). Using the trypan blue exclusion dye, cell death toll reached around 100% after 24- and 72-hour exposure to mancozeb 1 µM and 0.5 µM respectively. Reactive oxygen species generated by mancozeb, which peaked at 4 µM, could be the cause of cell death. The number and length of neurites were concentration-dependently reduced by mancozeb at sub-µM concentrations, and this was accompanied by changes in cell biomechanical characteristics (stiffness) as determined by atomic force microscopy. The uncertainty factor obtained from our cytotoxic studies, when performing risk assessment of mancozeb, varied from 200 to 2000, which may result in detectable neurotoxicity in humans in accordance with international regulatory agencies recommendations.

Keywords: Atomic force microscopy, cytotoxicity tests, mancozeb, neurons, oxidative stress, zinc

ANOVA OF MTT DATA (24 H)

```
> library(readxl)
> MTT_D1 <- read_excel("MTT_D1.xlsx", col_types = c("text",
+                                                     "text", "numeric", "numeric"))
> View(MTT_D1)
> attach(MTT_D1)
> names(MTT_D1)
[1] "Dose"            "Replicate"       "Response"        "Normalized_data"
> class("Dose")
[1] "character"
> factor(Dose)
 [1] 0  0  0  0  0  0  0  0  1  1  1  1  1  1  1  1  2  2  2  2  2  2  2  2  4  4  4  4
4  4  4
[32] 4  8  8  8  8  8  8  8  8  16 16 16 16 16 16 16 16
Levels: 0 1 16 2 4 8
> summary(MTT_D1)
     Dose              Replicate            Response          Normalized_data
 Length:48          Length:48          Min.   :-0.0003333   Min.   : -0.1096
 Class :character   Class :character   1st Qu.: 0.0844167   1st Qu.: 27.7686
 Mode  :character   Mode  :character   Median : 0.2533333   Median : 83.3333
                                       Mean   : 0.2075069   Mean   : 68.2589
                                       3rd Qu.: 0.2921667   3rd Qu.: 96.1075
                                       Max.   : 0.4083333   Max.   :134.3202
> aov(Normalized_data~Dose)
Call:
   aov(formula = Normalized_data ~ Dose)

Terms:
                    Dose Residuals
Sum of Squares  57244.38   8921.46
Deg. of Freedom        5        42

Residual standard error: 14.57449
Estimated effects may be unbalanced
> AOV_D1_MTT <- aov(Normalized_data~Dose)
> summary(AOV_D1_MTT)
            Df Sum Sq Mean Sq F value Pr(>F)
Dose         5  57244   11449    53.9 <2e-16 ***
Residuals   42   8921     212
---
Signif. codes:  0 ‘***’ 0.001 ‘**’ 0.01 ‘*’ 0.05 ‘.’ 0.1 ‘ ’ 1
> TukeyHSD(AOV_D1_MTT)
  Tukey multiple comparisons of means
    95% family-wise confidence level

Fit: aov(formula = Normalized_data ~ Dose)

$Dose
           diff         lwr         upr     p adj
1-0   -5.441338  -27.195576  16.3129002 0.9746853
16-0 -88.884320 -110.638558 -67.1300823 0.0000000
2-0   -6.661184  -28.415422  15.0930537 0.9407204
4-0  -20.805921  -42.560159   0.9483169 0.0680144
8-0  -69.723136  -91.477374 -47.9688980 0.0000000
16-1 -83.442982 -105.197220 -61.6887445 0.0000000
2-1   -1.219846  -22.974084  20.5343914 0.9999803
4-1  -15.364583  -37.118821   6.3896546 0.3027833
8-1  -64.281798  -86.036036 -42.5275603 0.0000000
2-16  82.223136   60.468898 103.9773739 0.0000000
4-16  68.078399   46.324161  89.8326370 0.0000000
8-16  19.161184   -2.593054  40.9154221 0.1124992
4-2  -14.144737  -35.898975   7.6095011 0.3925616
8-2  -63.061952  -84.816190 -41.3077138 0.0000000
8-4  -48.917215  -70.671453 -27.1629770 0.0000005
```

(72 H)

```
> MTT_D2 <- read_excel("MTT_D2.xlsx", col_types = c("text",
+                                                    "text", "numeric", "numeric"))
> View(MTT_D2)
> attach(MTT_D2)
> names(MTT_D2)
[1] "Dose"             "Replicate"        "Response"         "Normalized_data"
> class("Dose")
[1] "character"
> factor(Dose)
 [1] 0  0  0  0  0  0  0  0  1  1  1  1  1  1  1  1  2  2  2  2  2  2  2  2  4  4
4  4  4  4  4
[32] 4  8  8  8  8  8  8  8  8  16 16 16 16 16 16 16 16
Levels: 0 1 16 2 4 8
> summary(MTT_D2)
     Dose             Replicate            Response          Normalized_data
 Length:48          Length:48          Min.   :-0.01933   Min.   :  0.00
 Class :character   Class :character   1st Qu.: 0.02800   1st Qu.: 10.53
 Mode  :character   Mode  :character   Median : 0.07983   Median : 30.01
                                       Mean   : 0.11608   Mean   : 43.85
                                       3rd Qu.: 0.20350   3rd Qu.: 76.50
                                       Max.   : 0.30367   Max.   :114.16
> aov(Normalized_data~Dose)
Call:
   aov(formula = Normalized_data ~ Dose)

Terms:
                    Dose Residuals
Sum of Squares  60123.02   5037.03
Deg. of Freedom        5        42

Residual standard error: 10.95123
Estimated effects may be unbalanced
> AOV_D2_MTT <- aov(Normalized_data~Dose)
> summary(AOV_D2_MTT)
            Df Sum Sq Mean Sq F value Pr(>F)    
Dose         5  60123   12025   100.3 <2e-16 ***
Residuals   42   5037     120                   
---
Signif. codes:  0 ‘***’ 0.001 ‘**’ 0.01 ‘*’ 0.05 ‘.’ 0.1 ‘ ’ 1
> TukeyHSD(AOV_D2_MTT)
  Tukey multiple comparisons of means
    95% family-wise confidence level

Fit: aov(formula = Normalized_data ~ Dose)

$Dose
           diff        lwr        upr     p adj
1-0  -20.551378  -36.89745  -4.205308 0.0065765
16-0 -87.562657 -103.90873 -71.216586 0.0000000
2-0  -52.600251  -68.94632 -36.254180 0.0000000
4-0  -82.581454  -98.92752 -66.235383 0.0000000
8-0  -93.530702 -109.87677 -77.184632 0.0000000
16-1 -67.011278  -83.35735 -50.665208 0.0000000
2-1  -32.048872  -48.39494 -15.702802 0.0000092
4-1  -62.030075  -78.37615 -45.684005 0.0000000
8-1  -72.979323  -89.32539 -56.633253 0.0000000
2-16  34.962406   18.61634  51.308476 0.0000016
4-16   4.981203  -11.36487  21.327273 0.9418638
8-16  -5.968045  -22.31412  10.378025 0.8826461
4-2  -29.981203  -46.32727 -13.635133 0.0000318
8-2  -40.930451  -57.27652 -24.584381 0.0000000
8-4  -10.949248  -27.29532   5.396822 0.3597055
```

```
> library(readxl)
> MTT_D5 <- read_excel("MTT_D5.xlsx", col_types = c("text",
+                                                   "text", "numeric", "numeric"))
> View(MTT_D5)
> attach(MTT_D5)
> names(MTT_D5)
[1] "Dose"              "Replicate"         "Response"          "Normalized_data"
> class("Dose")
[1] "character"
> factor(Dose)
 [1] 0  0  0  0  0  0  0  0  1  1  1  1  1  1  1  1  2  2  2  2  2  2  2  2  4  4
4  4  4  4  4
[32] 4  8  8  8  8  8  8  8  8  16 16 16 16 16 16 16 16
Levels: 0 1 16 2 4 8
> summary(MTT_D5)
     Dose             Replicate             Response          Normalized_data
 Length:48          Length:48          Min.    :-0.044667   Min.    :-16.5432
 Class :character   Class :character   1st Qu.: 0.002417   1st Qu.:  0.8951
 Mode  :character   Mode  :character   Median : 0.111333   Median : 41.2346
                                       Mean   : 0.116021   Mean   : 42.9707
                                       3rd Qu.: 0.208000   3rd Qu.: 77.0370
                                       Max.   : 0.389667   Max.   :144.3210

> aov(Normalized_data~Dose)
Call:
   aov(formula = Normalized_data ~ Dose)

Terms:
                    Dose Residuals
Sum of Squares  89197.64  11396.98
Deg. of Freedom        5        42

Residual standard error: 16.4729
Estimated effects may be unbalanced
> AOV_D5_MTT <- aov(Normalized_data~Dose)
> summary(AOV_D5_MTT)
            Df Sum Sq Mean Sq F value Pr(>F)    
Dose         5  89198   17840   65.74 <2e-16 ***
Residuals   42  11397     271                   
---
Signif. codes:  0 ‘***’ 0.001 ‘**’ 0.01 ‘*’ 0.05 ‘.’ 0.1 ‘ ’ 1
> TukeyHSD(AOV_D5_MTT)
  Tukey multiple comparisons of means
    95% family-wise confidence level

Fit: aov(formula = Normalized_data ~ Dose)

$Dose
             diff        lwr        upr     p adj
1-0    -7.1450617  -31.73292  17.442798 0.9521490
16-0 -102.2993827 -126.88724 -77.711523 0.0000000
2-0   -40.9567901  -65.54465 -16.368931 0.0001610
4-0   -89.8456790 -114.43354 -65.257820 0.0000000
8-0  -102.0216049 -126.60946 -77.433746 0.0000000
16-1  -95.1543210 -119.74218 -70.566462 0.0000000
2-1   -33.8117284  -58.39959  -9.223869 0.0023622
4-1   -82.7006173 -107.28848 -58.112758 0.0000000
8-1   -94.8765432 -119.46440 -70.288684 0.0000000
2-16   61.3425926   36.75473  85.930452 0.0000000
4-16   12.4537037  -12.13416  37.041563 0.6586421
8-16    0.2777778  -24.31008  24.865637 1.0000000
4-2   -48.8888889  -73.47675 -24.301030 0.0000070
8-2   -61.0648148  -85.65267 -36.476955 0.0000001
8-4   -12.1759259  -36.76379  12.411933 0.6795473
```

MTT statistics 24 and 48 h

```
> tidy(LL.4Model_MTT_Total)
# A tibble: 8 × 6
  term       curve estimate std.error statistic    p.value
  <chr>      <chr>    <dbl>     <dbl>     <dbl>      <dbl>
1 Hill Slope A         3.55     0.636      5.58 0.00506
2 Hill Slope B         2.47     0.368      6.72 0.00255
3 Min        A         8.76     4.83       1.81 0.144
4 Min        B         8.14     2.80       2.91 0.0438
5 Max        A        97.0      2.30      42.1  0.00000191
6 Max        B        99.4      3.61      27.5  0.0000103
7 EC50       A         5.86      0.396     14.8  0.000121
8 EC50       B         1.73       0.129     13.5  0.000175

> compParm(LL.4Model_MTT_Total, "EC50", "-")

Comparison of parameter 'EC50'

    Estimate Std. Error t-value   p-value
A-B  4.12702    0.41634  9.9125 0.0005814 ***
---
Signif. codes:  0 ‘***’ 0.001 ‘**’ 0.01 ‘*’ 0.05 ‘.’ 0.1 ‘ ’ 1
```

ANALYSIS OF VARIANCE OF TRYPAN BLUE DATA

```
> library(readxl)
> Trypan_Blue_1_3_and_5_days_ <- read_excel("C:/Users/EVELIN CUADRO/Desktop/Datos MCZ Tox
icity/Trypan Blue (1 3 and 5 days).xlsx",
+     col_types = c("text", "numeric", "numeric",
+         "numeric"))

> View(Trypan_Blue_1_3_and_5_days_)
> attach(Trypan_Blue_1_3_and_5_days_)

> names(Trypan_Blue_1_3_and_5_days_)
[1] "Concentration"          "D1PercentageDeathCells" "D3PercentageDeathCells"
[4] "D5PercentageDeathCells"
> factor(Concentration)
 [1] 0.0µM 0.0µM 0.0µM 0.0µM 0.0µM 0.0µM 0.0µM 0.0µM 0.0µM 0.0µM 0.0µM 0.1µM 0.1µM
[14] 0.1µM 0.1µM 0.1µM 0.1µM 0.1µM 0.1µM 0.1µM 0.1µM 0.1µM 0.2µM 0.2µM 0.2µM 0.2µM
[27] 0.2µM 0.2µM 0.2µM 0.2µM 0.2µM 0.2µM 0.2µM 0.5µM 0.5µM 0.5µM 0.5µM 0.5µM 0.5µM
[40] 0.5µM 0.5µM 0.5µM 0.5µM 0.5µM 1µM   1µM   1µM   1µM   1µM   1µM   1µM   1µM
[53] 1µM   1µM   1µM   2µM   2µM   2µM   2µM   2µM   2µM   2µM   2µM   2µM   2µM
[66] 2µM
Levels: 0.0µM 0.1µM 0.2µM 0.5µM 1µM 2µM
> summary(Trypan_Blue_1_3_and_5_days_)
 Concentration      D1PercentageDeathCells D3PercentageDeathCells D5PercentageDeathCells
 Length:66          Min.   :  0.00         Min.   :  1.01         Min.   :  0.00
 Class :character   1st Qu.: 12.75         1st Qu.: 14.71         1st Qu.: 14.67
 Mode  :character   Median : 40.68         Median : 71.63         Median : 69.77
                    Mean   : 50.80         Mean   : 58.81         Mean   : 58.04
                    3rd Qu.:100.00         3rd Qu.:100.00         3rd Qu.:100.00
                    Max.   :100.00         Max.   :100.00         Max.   :100.00


 > aov(D1PercentageDeathCells~Concentration)
Call:
   aov(formula = D1PercentageDeathCells ~ Concentration)

Terms:
                Concentration Residuals
Sum of Squares       98121.37  10044.83
Deg. of Freedom             5        60

Residual standard error: 12.93885
Estimated effects may be unbalanced

> aov(D3PercentageDeathCells~Concentration)
Call:
   aov(formula = D3PercentageDeathCells ~ Concentration)

Terms:
                Concentration Residuals
Sum of Squares      106975.48   3594.88
Deg. of Freedom             5        60

Residual standard error: 7.740452
Estimated effects may be unbalanced

> aov(D5PercentageDeathCells~Concentration)
Call:
   aov(formula = D5PercentageDeathCells ~ Concentration)

Terms:
                Concentration Residuals
Sum of Squares      114958.39   1745.15
Deg. of Freedom             5        60

Residual standard error: 5.393131
Estimated effects may be unbalanced
> AOV_TB_D1 <- aov(D1PercentageDeathCells~Concentration)
> AOV_TB_D3 <- aov(D3PercentageDeathCells~Concentration)
```

```
> AOV_TB_D5 <- aov(D5PercentageDeathCells~Concentration)

> summary(AOV_TB_D1)
              Df Sum Sq Mean Sq F value Pr(>F)
Concentration  5  98121   19624   117.2 <2e-16 ***
Residuals     60  10045     167
---
Signif. codes:  0 ‘***’ 0.001 ‘**’ 0.01 ‘*’ 0.05 ‘.’ 0.1 ‘ ’ 1
> summary(AOV_TB_D3)
              Df Sum Sq Mean Sq F value Pr(>F)
Concentration  5 106975   21395   357.1 <2e-16 ***
Residuals     60   3595      60
---
Signif. codes:  0 ‘***’ 0.001 ‘**’ 0.01 ‘*’ 0.05 ‘.’ 0.1 ‘ ’ 1
> summary(AOV_TB_D5)
              Df Sum Sq Mean Sq F value Pr(>F)
Concentration  5 114958   22992   790.5 <2e-16 ***
Residuals     60   1745      29
---
Signif. codes:  0 ‘***’ 0.001 ‘**’ 0.01 ‘*’ 0.05 ‘.’ 0.1 ‘ ’ 1
```

```
 > boxplot(D1PercentageDeathCells~Concentration,
+         col = rgb(0.82, 0.93, 0.93, alpha = 0.4),
+         xlab = "[Mancozeb]",
+         ylab = "% of dead cells",
+         main = "24 Hours")
```

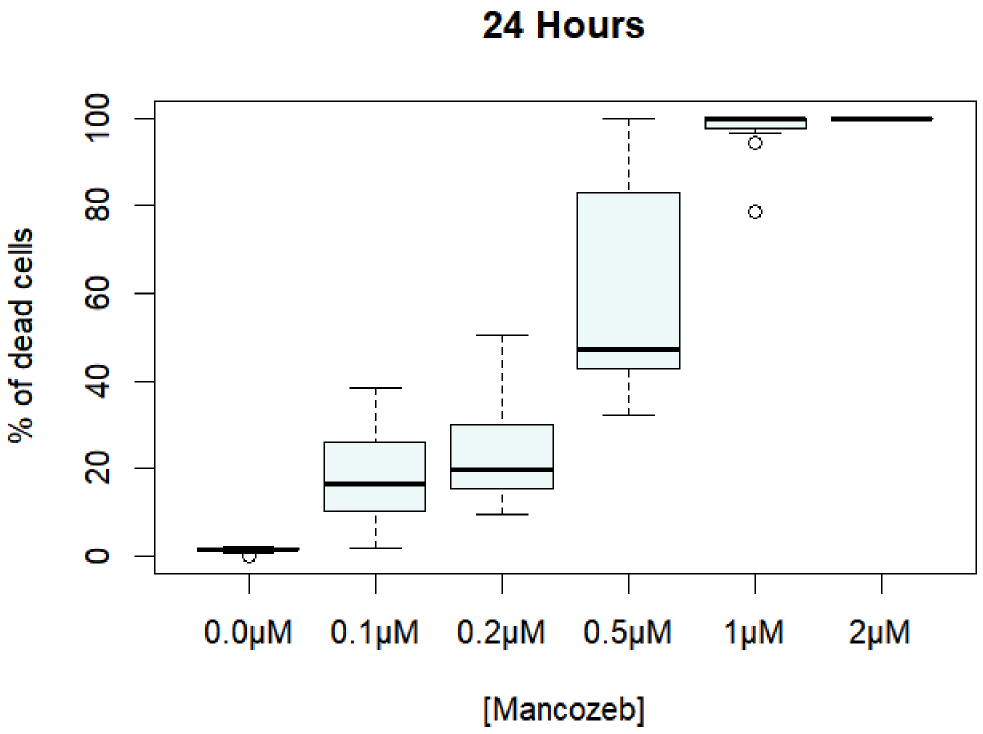


```
> boxplot(D3PercentageDeathCells~Concentration,
+         col = rgb(0.82, 0.93, 0.93, alpha = 0.4),
```

```
+          xlab = "[Mancozeb]",
+          ylab = "% of dead cells",
+          main = "72 Hours")
```

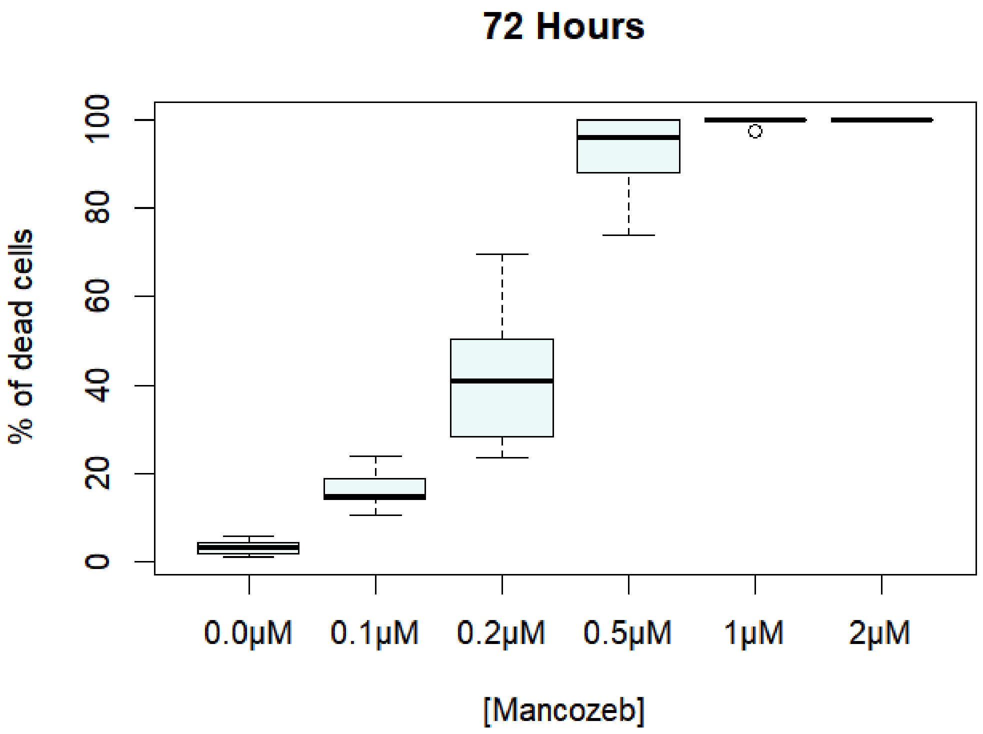


```
> boxplot(D5PercentageDeathCells~Concentration,
+          col = rgb(0.82, 0.93, 0.93, alpha = 0.4),
+          xlab = "[Mancozeb]",
+          ylab = "% of dead cells",
+          main = "120 Hours")
```

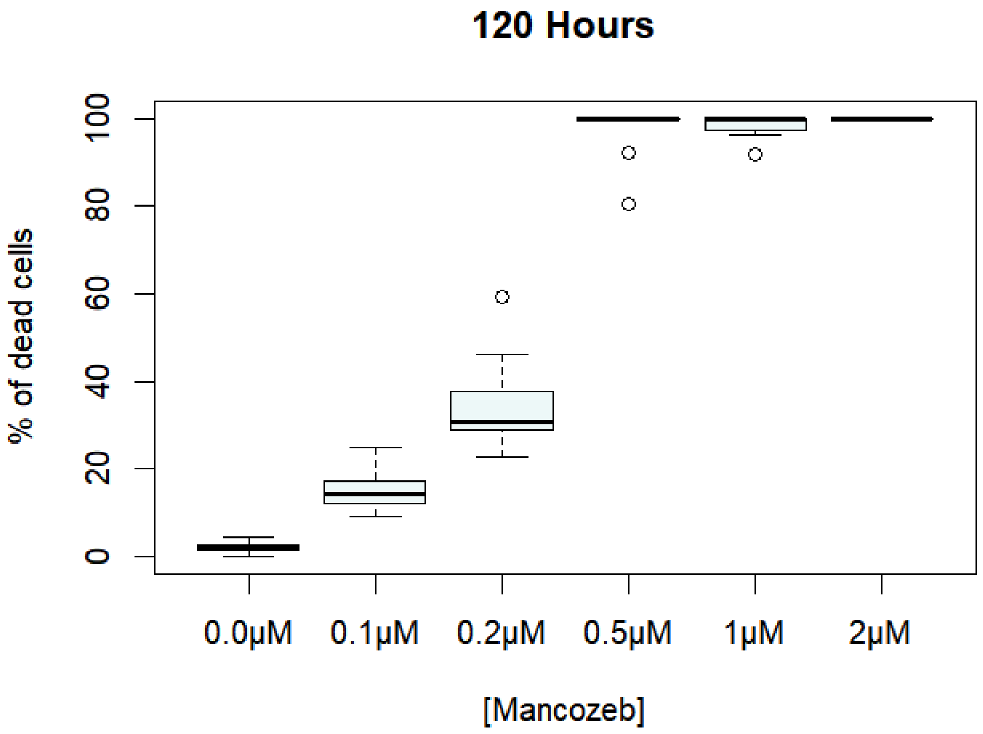

ANALYSIS OF VARIANCE: NUMBER OF NEURITES PER CELL.

Entre dosis

```
> library(readxl)
> NONeurites <- read_excel("C:/Users/EVELIN CUADRO/Desktop/Datos MCZ To
xicity/NONeurites.xlsx",
+     col_types = c("text", "numeric", "numeric",
+         "numeric"))
> View(NONeurites)
> attach(NONeurites)

> names(NONeurites)
[1] "Concentration" "D1NOneurites"  "D3NOneurites"  "D5NOneurites"

> class(Concentration)
[1] "character"
> class(D1NOneurites)
[1] "numeric"
> class(D3NOneurites)
[1] "numeric"
> class(D5NOneurites)
[1] "numeric"

> factor(Concentration)
 [1] 0.0µM 0.0µM 0.0µM 0.0µM 0.0µM 0.0µM 0.0µM 0.0µM 0.0µM 0.0µM 0.0µM
[12] 0.1µM 0.1µM 0.1µM 0.1µM 0.1µM 0.1µM 0.1µM 0.1µM 0.1µM 0.1µM 0.1µM
[23] 0.2µM 0.2µM 0.2µM 0.2µM 0.2µM 0.2µM 0.2µM 0.2µM 0.2µM 0.2µM 0.2µM
[34] 0.5µM 0.5µM 0.5µM 0.5µM 0.5µM 0.5µM 0.5µM 0.5µM 0.5µM 0.5µM 0.5µM
[45] 1µM   1µM   1µM   1µM   1µM   1µM   1µM   1µM   1µM   1µM   1µM
[56] 2µM   2µM   2µM   2µM   2µM   2µM   2µM   2µM   2µM   2µM   2µM
Levels: 0.0µM 0.1µM 0.2µM 0.5µM 1µM 2µM

> summary(NONeurites)
 Concentration        D1NOneurites      D3NOneurites      D5NOneurites
 Length:66          Min.   :0.00000   Min.   :0.00000   Min.   :0.00000
 Class :character   1st Qu.:0.06773   1st Qu.:0.05474   1st Qu.:0.08546
 Mode  :character   Median :0.86859   Median :1.09704   Median :1.29167
                    Mean   :1.38853   Mean   :1.65379   Mean   :1.35229
                    3rd Qu.:2.47177   3rd Qu.:3.04846   3rd Qu.:2.44949
                    Max.   :5.93750   Max.   :5.33333   Max.   :4.37037


> boxplot(D1NOneurites~Concentration)
```

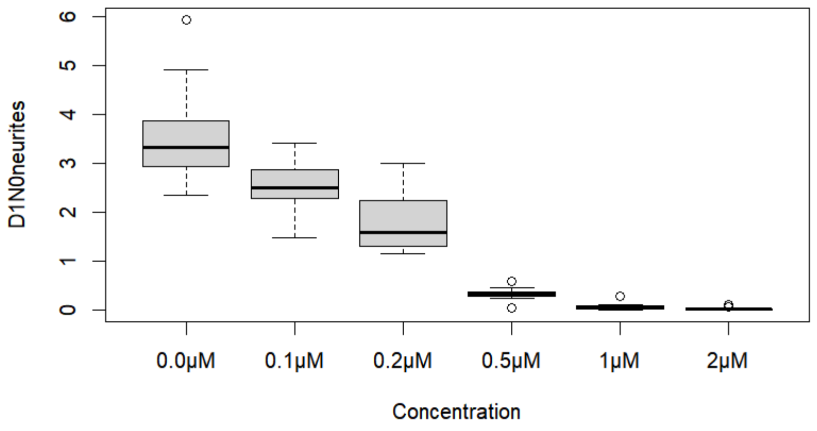

```
> boxplot(D3N0neurites~Concentration)
```

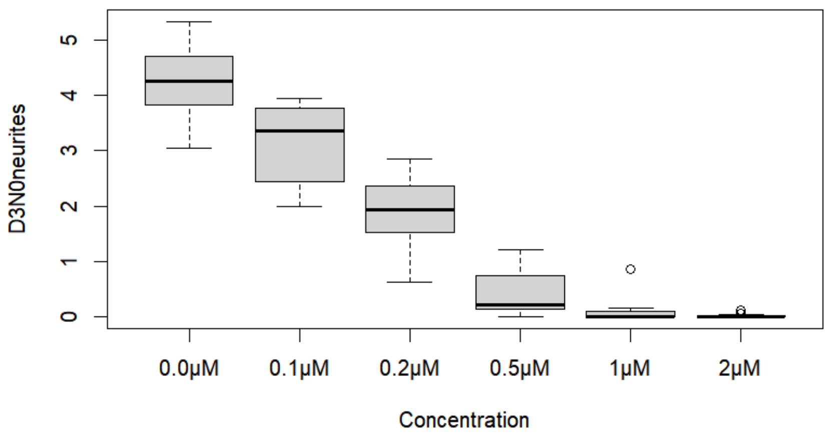


```
> boxplot(D5N0neurites~Concentration)
```

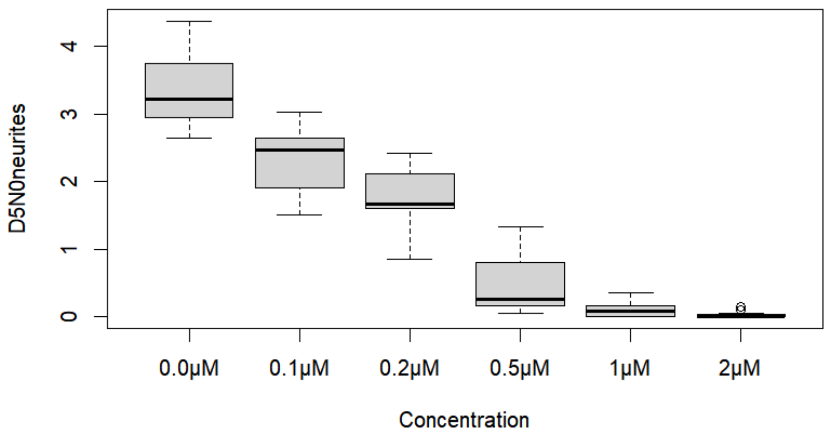

```
> aov(D1N0neurites~Concentration)

Call:
   aov(formula = D1N0neurites ~ Concentration)

Terms:
                Concentration Residuals
Sum of Squares      121.26018  17.76454
Deg. of Freedom             5        60

Residual standard error: 0.5441284
Estimated effects may be unbalanced


> aov(D3N0neurites~Concentration)
Call:
   aov(formula = D3N0neurites ~ Concentration)

Terms:
                Concentration Residuals
Sum of Squares      172.04333  17.29542
Deg. of Freedom             5        60

Residual standard error: 0.5368957
Estimated effects may be unbalanced


> aov(D5N0neurites~Concentration)
Call:
   aov(formula = D5N0neurites ~ Concentration)

Terms:
                Concentration Residuals
Sum of Squares      101.46431   9.86062
Deg. of Freedom             5        60

Residual standard error: 0.4053933
Estimated effects may be unbalanced



> AOV_D1_NN <- aov(D1N0neurites~Concentration) #Anova del día 1
> AOV_D3_NN <- aov(D3N0neurites~Concentration) #Anova del día 3
> AOV_D5_NN <- aov(D5N0neurites~Concentration) #Anova del día 5


> summary(AOV_D1_NN)
              Df Sum Sq Mean Sq F value Pr(>F)
Concentration  5 121.26  24.252   81.91 <2e-16 ***
Residuals     60  17.76   0.296
---
Signif. codes:  0 ‘***’ 0.001 ‘**’ 0.01 ‘*’ 0.05 ‘.’ 0.1 ‘ ’ 1


> summary(AOV_D3_NN)
              Df Sum Sq Mean Sq F value Pr(>F)
Concentration  5  172.0   34.41   119.4 <2e-16 ***
Residuals     60   17.3    0.29
---
Signif. codes:  0 ‘***’ 0.001 ‘**’ 0.01 ‘*’ 0.05 ‘.’ 0.1 ‘ ’ 1


> summary(AOV_D5_NN)
              Df Sum Sq Mean Sq F value Pr(>F)
Concentration  5 101.46  20.293   123.5 <2e-16 ***
Residuals     60   9.86   0.164
---
Signif. codes:  0 ‘***’ 0.001 ‘**’ 0.01 ‘*’ 0.05 ‘.’ 0.1 ‘ ’ 1
```

```
> TukeyHSD(AOV_D1_NN)
  Tukey multiple comparisons of means
    95% family-wise confidence level

Fit: aov(formula = D1NOneurites ~ Concentration)

$Concentration
                   diff        lwr         upr     p adj
0.1µM-0.0µM -1.06730724 -1.7503191 -0.38429542 0.0003111
0.2µM-0.0µM -1.82555253 -2.5085644 -1.14254071 0.0000000
0.5µM-0.0µM -3.26778151 -3.9507933 -2.58476969 0.0000000
1µM-0.0µM   -3.52290188 -4.2059137 -2.83989006 0.0000000
2µM-0.0µM   -3.56772068 -4.2507325 -2.88470886 0.0000000
0.2µM-0.1µM -0.75824529 -1.4412571 -0.07523347 0.0211499
0.5µM-0.1µM -2.20047426 -2.8834861 -1.51746245 0.0000000
1µM-0.1µM   -2.45559464 -3.1386065 -1.77258282 0.0000000
2µM-0.1µM   -2.50041343 -3.1834253 -1.81740161 0.0000000
0.5µM-0.2µM -1.44222897 -2.1252408 -0.75921715 0.0000008
1µM-0.2µM   -1.69734935 -2.3803612 -1.01433753 0.0000000
2µM-0.2µM   -1.74216814 -2.4251800 -1.05915632 0.0000000
1µM-0.5µM   -0.25512038 -0.9381322  0.42789144 0.8796328
2µM-0.5µM   -0.29993917 -0.9829510  0.38307265 0.7877997
2µM-1µM     -0.04481879 -0.7278306  0.63819303 0.9999609

> TukeyHSD(AOV_D3_NN)
  Tukey multiple comparisons of means
    95% family-wise confidence level

Fit: aov(formula = D3NOneurites ~ Concentration)

$Concentration
                   diff        lwr        upr     p adj
0.1µM-0.0µM -1.15406132 -1.8279943 -0.4801283 0.0000648
0.2µM-0.0µM -2.39648696 -3.0704200 -1.7225539 0.0000000
0.5µM-0.0µM -3.82550314 -4.4994362 -3.1515701 0.0000000
1µM-0.0µM   -4.17127239 -4.8452054 -3.4973394 0.0000000
2µM-0.0µM   -4.26888657 -4.9428196 -3.5949535 0.0000000
0.2µM-0.1µM -1.24242564 -1.9163587 -0.5684926 0.0000157
0.5µM-0.1µM -2.67144182 -3.3453749 -1.9975088 0.0000000
1µM-0.1µM   -3.01721107 -3.6911441 -2.3432780 0.0000000
2µM-0.1µM   -3.11482526 -3.7887583 -2.4408922 0.0000000
0.5µM-0.2µM -1.42901618 -2.1029492 -0.7550831 0.0000007
1µM-0.2µM   -1.77478542 -2.4487185 -1.1008524 0.0000000
2µM-0.2µM   -1.87239961 -2.5463326 -1.1984666 0.0000000
1µM-0.5µM   -0.34576925 -1.0197023  0.3281638 0.6590596
2µM-0.5µM   -0.44338344 -1.1173165  0.2305496 0.3905647
2µM-1µM     -0.09761419 -0.7715472  0.5763188 0.9981178

> TukeyHSD(AOV_D5_NN)
  Tukey multiple comparisons of means
    95% family-wise confidence level

Fit: aov(formula = D5NOneurites ~ Concentration)

$Concentration
                   diff        lwr         upr     p adj
0.1µM-0.0µM -1.05100160 -1.5598675 -0.54213566 0.0000013
0.2µM-0.0µM -1.60865556 -2.1175215 -1.09978962 0.0000000
0.5µM-0.0µM -2.86479523 -3.3736612 -2.35592929 0.0000000
1µM-0.0µM   -3.27201700 -3.7808829 -2.76315107 0.0000000
2µM-0.0µM   -3.34450526 -3.8533712 -2.83563932 0.0000000
0.2µM-0.1µM -0.55765396 -1.0665199 -0.04878803 0.0237512
0.5µM-0.1µM -1.81379363 -2.3226596 -1.30492770 0.0000000
1µM-0.1µM   -2.22101540 -2.7298813 -1.71214947 0.0000000
2µM-0.1µM   -2.29350366 -2.8023696 -1.78463773 0.0000000
0.5µM-0.2µM -1.25613967 -1.7650056 -0.74727373 0.0000000
1µM-0.2µM   -1.66336144 -2.1722274 -1.15449551 0.0000000
2µM-0.2µM   -1.73584970 -2.2447156 -1.22698376 0.0000000
1µM-0.5µM   -0.40722177 -0.9160877  0.10164417 0.1886670
2µM-0.5µM   -0.47971003 -0.9885760  0.02915591 0.0756219
2µM-1µM     -0.07248826 -0.5813542  0.43637768 0.9982620
```

Entre días

```
> library(readxl)
> NONeurites_by_day_ <- read_excel("C:/Users/EVELIN CUADRO/Desktop/Dato
s MCZ Toxicity/NONeurites (by day).xlsx",
+     col_types = c("text", "numeric", "numeric",
+         "numeric", "numeric", "numeric",
+         "numeric"))
> View(NONeurites_by_day_)
> attach(NONeurites_by_day_)
> names(NONeurites_by_day_)
[1] "Day"     "Control" "C1"      "C2"      "C3"      "C4"      "C5"
> class(Day)
[1] "character"
> class(Control)
[1] "numeric"
> class(C1)
[1] "numeric"
> class(C2)
[1] "numeric"
> class(C3)
[1] "numeric"
> class(C4)
[1] "numeric"
> class(C5)
[1] "numeric"
> factor(Day)
 [1] 1 1 1 1 1 1 1 1 1 1 1 3 3 3 3 3 3 3 3 3 3 3 5 5 5 5 5 5 5 5 5 5 5
Levels: 1 3 5

> boxplot(Control~Day)
```

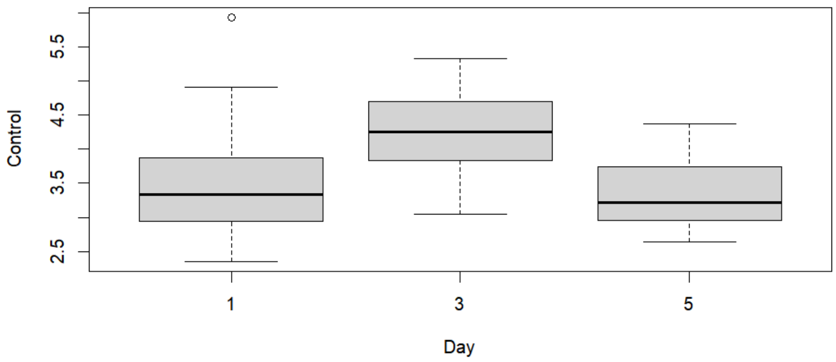


```
> boxplot(C1~Day)
```

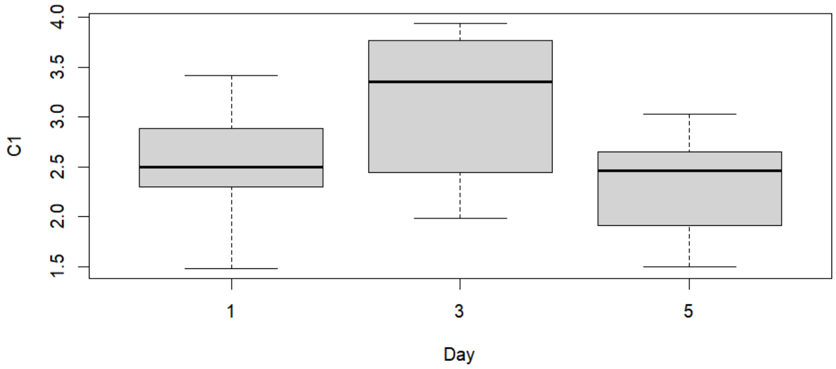

```
> boxplot(C2~Day)
```

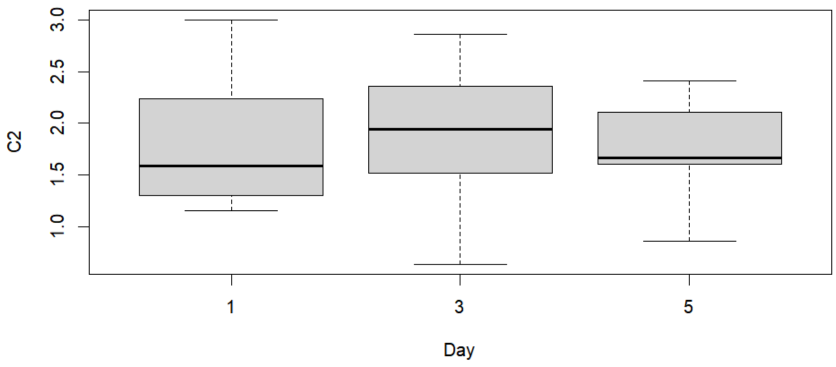


```
> boxplot(C3~Day)
```

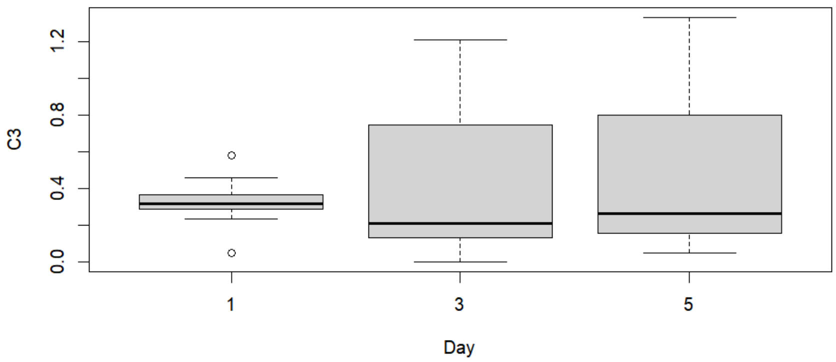


```
> boxplot(C4~Day)
```

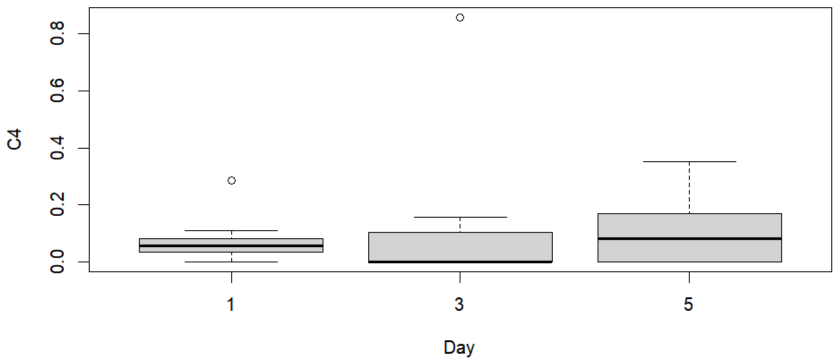

```
> boxplot(C5~Day)
```

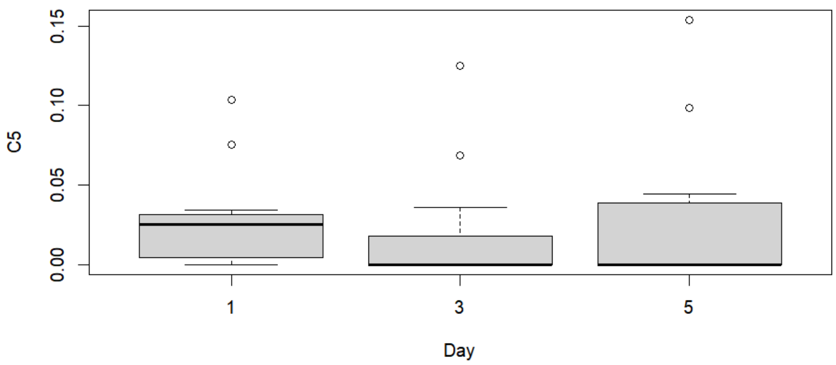


```
> aov(Control~Day)
Call:
   aov(formula = Control ~ Day)

Terms:
                     Day Residuals
Sum of Squares   5.002561 19.278700
Deg. of Freedom         2        30

Residual standard error: 0.8016379
Estimated effects may be unbalanced

> aov(C1~Day)
Call:
   aov(formula = C1 ~ Day)

Terms:
                     Day Residuals
Sum of Squares   3.912089 10.094430
Deg. of Freedom         2        30

Residual standard error: 0.5800698
Estimated effects may be unbalanced

> aov(C2~Day)
Call:
   aov(formula = C2 ~ Day)

Terms:
                     Day Residuals
Sum of Squares   0.112882 10.218817
Deg. of Freedom         2        30

Residual standard error: 0.5836328
Estimated effects may be unbalanced

> aov(C3~Day)
Call:
   aov(formula = C3 ~ Day)

Terms:
                    Day Residuals
Sum of Squares  0.195887  4.424007
Deg. of Freedom        2        30

Residual standard error: 0.3840142
Estimated effects may be unbalanced
```

```
> aov(C4~Day)
Call:
   aov(formula = C4 ~ Day)

Terms:
                      Day Residuals
Sum of Squares  0.0112338 0.8511000
Deg. of Freedom         2        30

Residual standard error: 0.168434
Estimated effects may be unbalanced

> aov(C5~Day)
Call:
   aov(formula = C5 ~ Day)

Terms:
                       Day  Residuals
Sum of Squares  0.00066568 0.05352666
Deg. of Freedom          2          30

Residual standard error: 0.04224005
Estimated effects may be unbalanced


> AOV_CONTROL_NN <- aov(Control~Day)
> AOV_C1_NN <- aov(C1~Day)
> AOV_C2_NN <- aov(C2~Day)
> AOV_C3_NN <- aov(C3~Day)
> AOV_C4_NN <- aov(C4~Day)
> AOV_C5_NN <- aov(C5~Day)


> summary(AOV_CONTROL_NN)
            Df Sum Sq Mean Sq F value Pr(>F)
Day          2  5.003  2.5013   3.892 0.0314 *
Residuals   30 19.279  0.6426
---
Signif. codes:  0 '***' 0.001 '**' 0.01 '*' 0.05 '.' 0.1 ' ' 1

> summary(AOV_C1_NN)
            Df Sum Sq Mean Sq F value  Pr(>F)
Day          2  3.912  1.9560   5.813 0.00735 **
Residuals   30 10.094  0.3365
---
Signif. codes:  0 '***' 0.001 '**' 0.01 '*' 0.05 '.' 0.1 ' ' 1

> summary(AOV_C2_NN)
            Df Sum Sq Mean Sq F value Pr(>F)
Day          2  0.113  0.0564   0.166  0.848
Residuals   30 10.219  0.3406

> summary(AOV_C3_NN)
            Df Sum Sq Mean Sq F value Pr(>F)
Day          2  0.196 0.09794   0.664  0.522
Residuals   30  4.424 0.14747

> summary(AOV_C4_NN)
            Df Sum Sq  Mean Sq F value Pr(>F)
Day          2 0.0112 0.005617   0.198  0.821
Residuals   30 0.8511 0.028370

> summary(AOV_C5_NN)
            Df  Sum Sq   Mean Sq F value Pr(>F)
Day          2 0.00067 0.0003328   0.187  0.831
Residuals   30 0.05353 0.0017842
```

Comentado [EGCB1]: Resumen de ANOVAs. Comparación entre los datos recolectado a los 1, 3 y 5 días post tratamiento en cada concentración

ANALYSIS OF VARIANCE OF NEURITE LENGTH PER CELL

Entre dosis

```
> library(readxl)
> NeuritesLength_by_dose_ <- read_excel("C:/Users/EVELIN CUADRO/Desktop/Datos MCZ T
oxicity/NeuritesLength (by dose).xlsx",
+     col_types = c("text", "numeric", "numeric",
+         "numeric"))

> View(NeuritesLength_by_dose_)
> attach(NeuritesLength_by_dose_)

> names(NeuritesLength_by_dose_)
[1] "Concentration"   "D1NeuriteLength" "D3NeuriteLength" "D5NeuriteLength"
> class(Concentration)
[1] "character"
> class(D1NeuriteLength)
[1] "numeric"
> class(D3NeuriteLength)
[1] "numeric"
> class(D5NeuriteLength)
[1] "numeric"

> factor(Concentration)
 [1] 0.0µM 0.0µM 0.0µM 0.0µM 0.0µM 0.0µM 0.0µM 0.0µM 0.0µM 0.0µM 0.0µM 0.1µM 0.1µM
[14] 0.1µM 0.1µM 0.1µM 0.1µM 0.1µM 0.1µM 0.1µM 0.1µM 0.1µM 0.2µM 0.2µM 0.2µM 0.2µM
[27] 0.2µM 0.2µM 0.2µM 0.2µM 0.2µM 0.2µM 0.2µM 0.5µM 0.5µM 0.5µM 0.5µM 0.5µM 0.5µM
[40] 0.5µM 0.5µM 0.5µM 0.5µM 0.5µM 1µM   1µM   1µM   1µM   1µM   1µM   1µM   1µM
[53] 1µM   1µM   1µM   2µM   2µM   2µM   2µM   2µM   2µM   2µM   2µM   2µM   2µM
[66] 2µM
Levels: 0.0µM 0.1µM 0.2µM 0.5µM 1µM 2µM

> summary(NeuritesLength_by_dose_)
 Concentration       D1NeuriteLength   D3NeuriteLength    D5NeuriteLength
 Length:66          Min.   : 0.0000   Min.   :  0.0000   Min.   :  0.0000
 Class :character   1st Qu.: 0.7218   1st Qu.:  0.5035   1st Qu.:  0.9337
 Mode  :character   Median :16.1316   Median : 16.8028   Median : 20.3286
                    Mean   :24.3924   Mean   : 31.5770   Mean   : 25.5528
                    3rd Qu.:42.7449   3rd Qu.: 56.1183   3rd Qu.: 44.6875
                    Max.   :96.5118   Max.   :104.4726   Max.   :100.5488

> boxplot(D1NeuriteLength~Concentration)
```

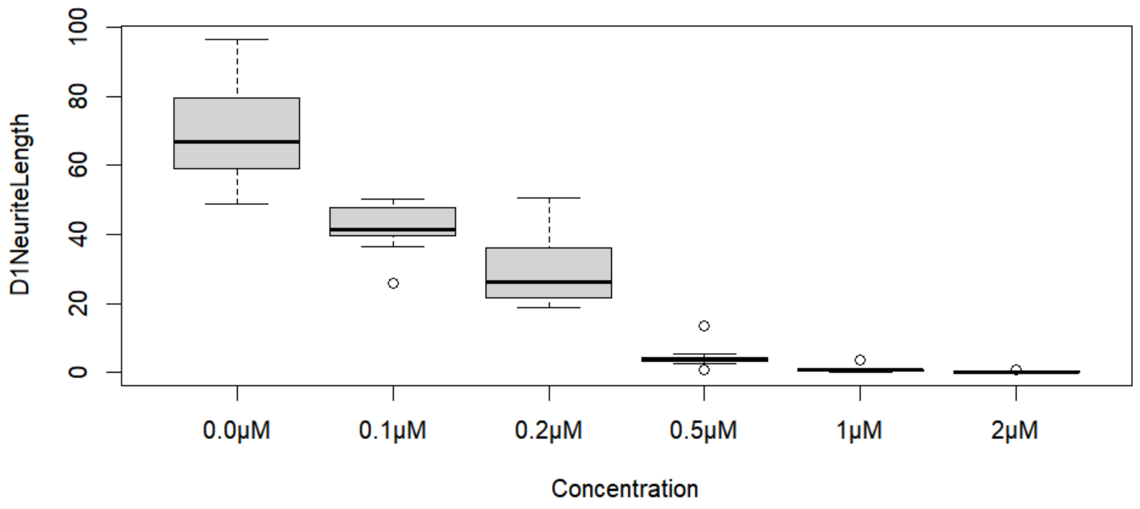

```
> boxplot(D3NeuriteLength~Concentration)
```

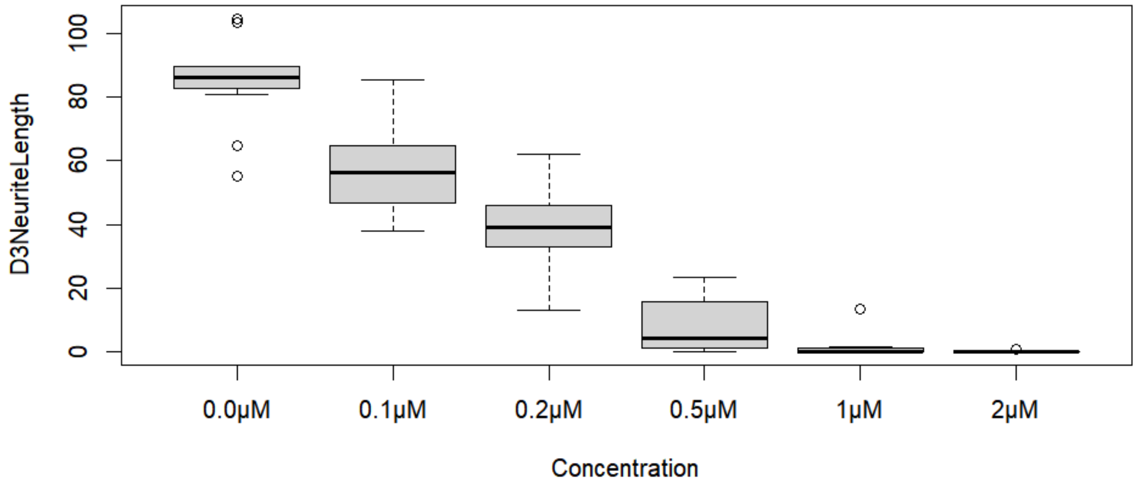


```
> boxplot(D5NeuriteLength~Concentration)
```

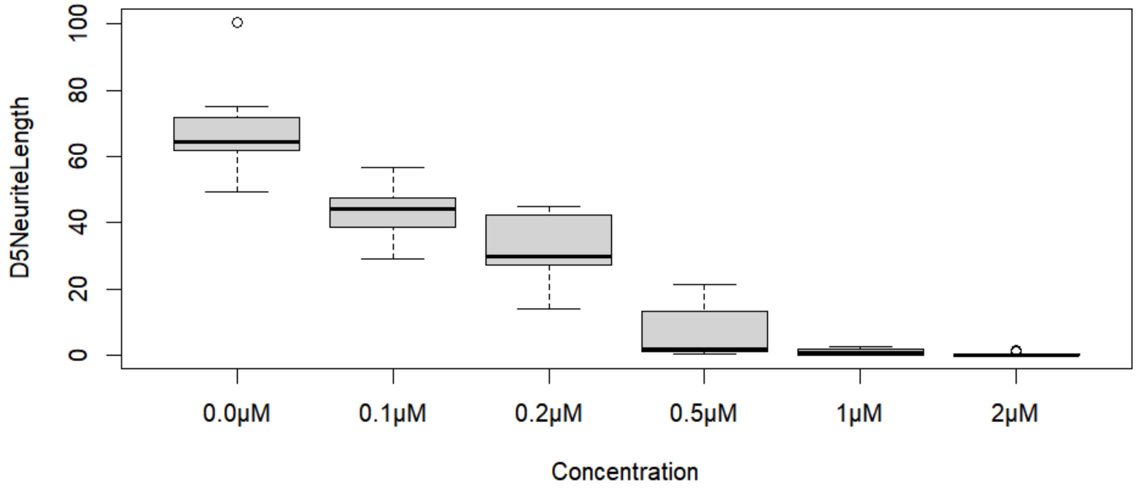


```
> aov(D1NeuriteLength~Concentration)
Call:
   aov(formula = D1NeuriteLength ~ Concentration)

Terms:
                Concentration Residuals
Sum of Squares       43322.96   4319.14
Deg. of Freedom             5        60

Residual standard error: 8.484436
Estimated effects may be unbalanced
```

```
> aov(D3NeuriteLength~Concentration)
Call:
   aov(formula = D3NeuriteLength ~ Concentration)

Terms:
                Concentration Residuals
Sum of Squares       65248.80   6905.63
Deg. of Freedom             5        60

Residual standard error: 10.72818
Estimated effects may be unbalanced
> aov(D5NeuriteLength~Concentration)
Call:
   aov(formula = D5NeuriteLength ~ Concentration)

Terms:
                Concentration Residuals
Sum of Squares       41419.06   3969.16
Deg. of Freedom             5        60

Residual standard error: 8.133433
Estimated effects may be unbalanced


> AOV_D1_NL <- aov(D1NeuriteLength~Concentration)
> AOV_D3_NL <- aov(D3NeuriteLength~Concentration)
> AOV_D5_NL <- aov(D5NeuriteLength~Concentration)

> summary(AOV_D1_NL)
              Df Sum Sq Mean Sq F value Pr(>F)
Concentration  5  43323    8665   120.4 <2e-16 ***
Residuals     60   4319      72
---
Signif. codes:  0 ‘***’ 0.001 ‘**’ 0.01 ‘*’ 0.05 ‘.’ 0.1 ‘ ’ 1
> summary(AOV_D3_NL)
              Df Sum Sq Mean Sq F value Pr(>F)
Concentration  5  65249   13050   113.4 <2e-16 ***
Residuals     60   6906     115
---
Signif. codes:  0 ‘***’ 0.001 ‘**’ 0.01 ‘*’ 0.05 ‘.’ 0.1 ‘ ’ 1
> summary(AOV_D5_NL)
              Df Sum Sq Mean Sq F value Pr(>F)
Concentration  5  41419    8284   125.2 <2e-16 ***
Residuals     60   3969      66
---
Signif. codes:  0 ‘***’ 0.001 ‘**’ 0.01 ‘*’ 0.05 ‘.’ 0.1 ‘ ’ 1

> TukeyHSD(AOV_D1_NL)
  Tukey multiple comparisons of means
    95% family-wise confidence level

Fit: aov(formula = D1NeuriteLength ~ Concentration)

$Concentration
                   diff       lwr        upr     p adj
0.1µM-0.0µM -27.9586305 -38.60864 -17.308625 0.0000000
0.2µM-0.0µM -40.7981764 -51.44818 -30.148171 0.0000000
0.5µM-0.0µM -65.5137512 -76.16376 -54.863746 0.0000000
1µM-0.0µM   -69.0429465 -79.69295 -58.392941 0.0000000
2µM-0.0µM   -69.6572643 -80.30727 -59.007259 0.0000000
0.2µM-0.1µM -12.8395459 -23.48955  -2.189541 0.0094360
0.5µM-0.1µM -37.5551207 -48.20513 -26.905116 0.0000000
1µM-0.1µM   -41.0843160 -51.73432 -30.434311 0.0000000
2µM-0.1µM   -41.6986338 -52.34864 -31.048629 0.0000000
0.5µM-0.2µM -24.7155748 -35.36558 -14.065570 0.0000001
1µM-0.2µM   -28.2447701 -38.89478 -17.594765 0.0000000
2µM-0.2µM   -28.8590879 -39.50909 -18.209083 0.0000000
1µM-0.5µM    -3.5291952 -14.17920   7.120810 0.9239297
2µM-0.5µM    -4.1435130 -14.79352   6.506492 0.8602876
2µM-1µM      -0.6143178 -11.26432  10.035687 0.9999793
```

```
> TukeyHSD(AOV_D3_NL)
  Tukey multiple comparisons of means
    95% family-wise confidence level

Fit: aov(formula = D3NeuriteLength ~ Concentration)

$Concentration
                  diff       lwr        upr     p adj
0.1µM-0.0µM -27.561682 -41.02812 -14.095240 0.0000016
0.2µM-0.0µM -46.791864 -60.25831 -33.325422 0.0000000
0.5µM-0.0µM -76.456802 -89.92324 -62.990360 0.0000000
1µM-0.0µM   -82.973280 -96.43972 -69.506838 0.0000000
2µM-0.0µM   -84.439618 -97.90606 -70.973176 0.0000000
0.2µM-0.1µM -19.230182 -32.69662  -5.763739 0.0011986
0.5µM-0.1µM -48.895120 -62.36156 -35.428678 0.0000000
1µM-0.1µM   -55.411598 -68.87804 -41.945156 0.0000000
2µM-0.1µM   -56.877936 -70.34438 -43.411493 0.0000000
0.5µM-0.2µM -29.664939 -43.13138 -16.198496 0.0000003
1µM-0.2µM   -36.181416 -49.64786 -22.714974 0.0000000
2µM-0.2µM   -37.647754 -51.11420 -24.181312 0.0000000
1µM-0.5µM    -6.516478 -19.98292   6.949965 0.7121526
2µM-0.5µM    -7.982815 -21.44926   5.483627 0.5083065
2µM-1µM      -1.466338 -14.93278  12.000105 0.9995263

> TukeyHSD(AOV_D5_NL)
  Tukey multiple comparisons of means
    95% family-wise confidence level

Fit: aov(formula = D5NeuriteLength ~ Concentration)

$Concentration
                   diff       lwr        upr     p adj
0.1µM-0.0µM -24.2383197 -34.44773 -14.028907 0.0000000
0.2µM-0.0µM -35.4232415 -45.63265 -25.213829 0.0000000
0.5µM-0.0µM -60.6369276 -70.84634 -50.427515 0.0000000
1µM-0.0µM   -67.1477043 -77.35712 -56.938292 0.0000000
2µM-0.0µM   -67.7804434 -77.98986 -57.571031 0.0000000
0.2µM-0.1µM -11.1849218 -21.39433  -0.975509 0.0238139
0.5µM-0.1µM -36.3986078 -46.60802 -26.189195 0.0000000
1µM-0.1µM   -42.9093846 -53.11880 -32.699972 0.0000000
2µM-0.1µM   -43.5421237 -53.75154 -33.332711 0.0000000
0.5µM-0.2µM -25.2136860 -35.42310 -15.004273 0.0000000
1µM-0.2µM   -31.7244628 -41.93388 -21.515050 0.0000000
2µM-0.2µM   -32.3572019 -42.56661 -22.147789 0.0000000
1µM-0.5µM    -6.5107768 -16.72019   3.698636 0.4258606
2µM-0.5µM    -7.1435159 -17.35293   3.065897 0.3221123
2µM-1µM      -0.6327391 -10.84215   9.576674 0.9999705
```

Entre días

```
> library(readxl)
> NeuritesLength_by_day_ <- read_excel("C:/Users/EVELIN CUADRO/Desktop/Datos MCZ To
xicity/NeuritesLength (by day).xlsx",
+     col_types = c("text", "numeric", "numeric",
+         "numeric", "numeric", "numeric",
+         "numeric"))

> View(NeuritesLength_by_day_)
> attach(NeuritesLength_by_day_)

> names(NeuritesLength_by_day_)
[1] "Day"     "Control" "C1"      "C2"      "C3"      "C4"      "C5"
> factor(Day)
 [1] 1 1 1 1 1 1 1 1 1 1 1 3 3 3 3 3 3 3 3 3 3 3 5 5 5 5 5 5 5 5 5 5 5
Levels: 1 3 5
> class(Control)
[1] "numeric"
> class(Day)
[1] "character"
> class(C1)
[1] "numeric"
> class(C2)
[1] "numeric"
> class(C3)
[1] "numeric"
> class(C4)
[1] "numeric"
> class(C5)
[1] "numeric"

> summary(NeuritesLength_by_day_)
     Day                Control             C1              C2
 Length:33          Min.   : 48.90   Min.   :25.93   Min.   :13.25
 Class :character   1st Qu.: 62.31   1st Qu.:40.58   1st Qu.:25.23
 Mode  :character   Median : 69.04   Median :46.10   Median :32.79
                    Mean   : 74.20   Mean   :47.61   Mean   :33.19
                    3rd Qu.: 89.02   3rd Qu.:50.18   3rd Qu.:42.91
                    Max.   :104.47   Max.   :85.30   Max.   :62.00
       C3               C4                C5
 Min.   : 0.000   Min.   : 0.0000   Min.   :0.0000
 1st Qu.: 1.463   1st Qu.: 0.0000   1st Qu.:0.0000
 Median : 3.853   Median : 0.6454   Median :0.0000
 Mean   : 6.662   Mean   : 1.1428   Mean   :0.2383
 3rd Qu.: 9.242   3rd Qu.: 1.0523   3rd Qu.:0.2856
 Max.   :23.260   Max.   :13.4835   Max.   :1.3788

> boxplot(Control~Day)
```

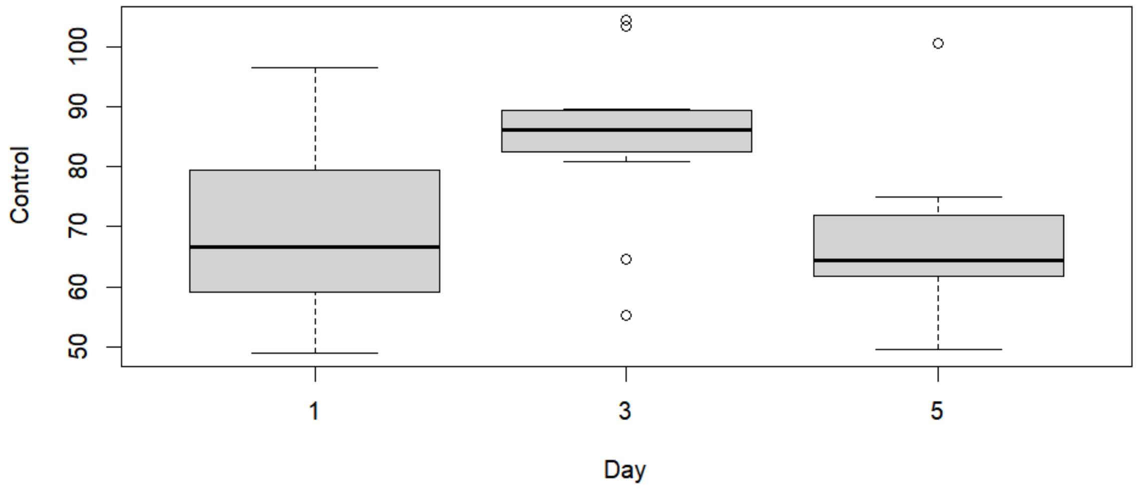

```
> boxplot(C1~Day)
```

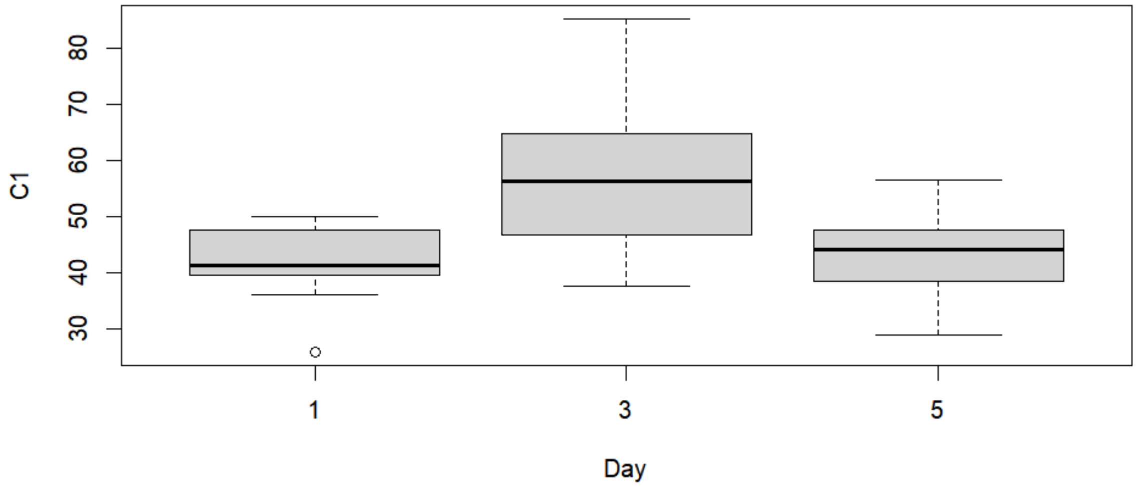


```
> boxplot(C2~Day)
```

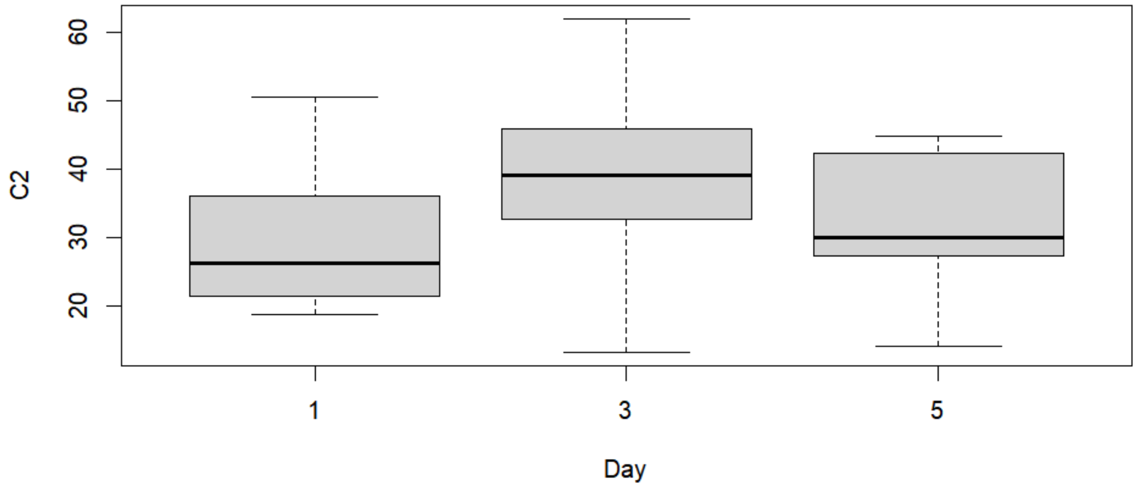


```
> boxplot(C3~Day)
```

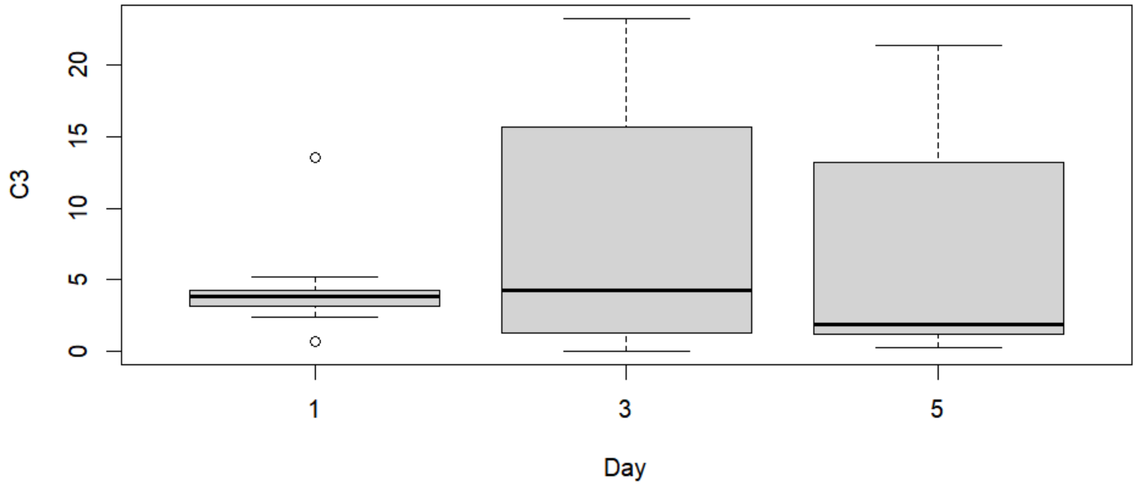

```
> boxplot(C4~Day)
```

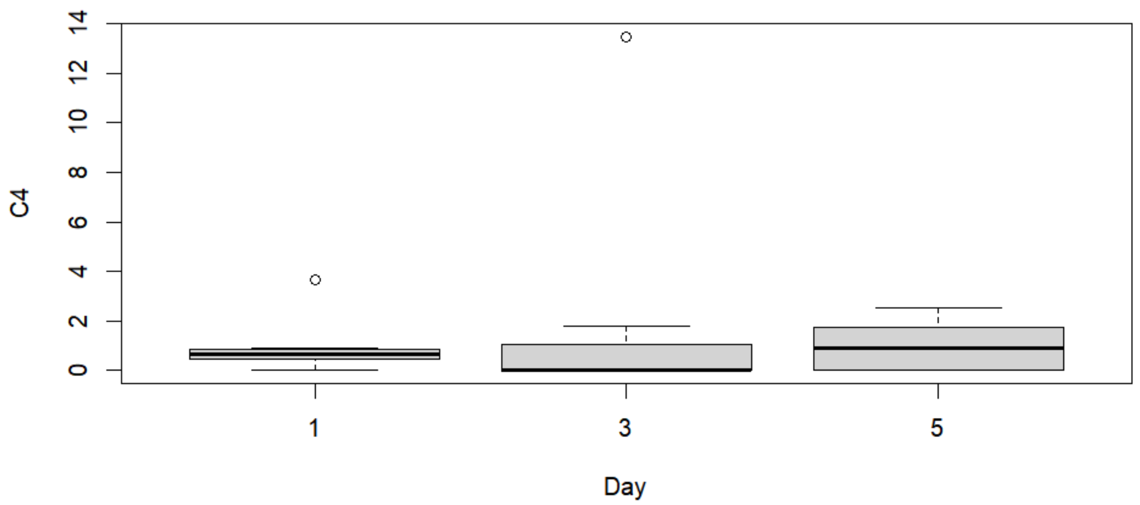


```
> boxplot(C5~Day)
```

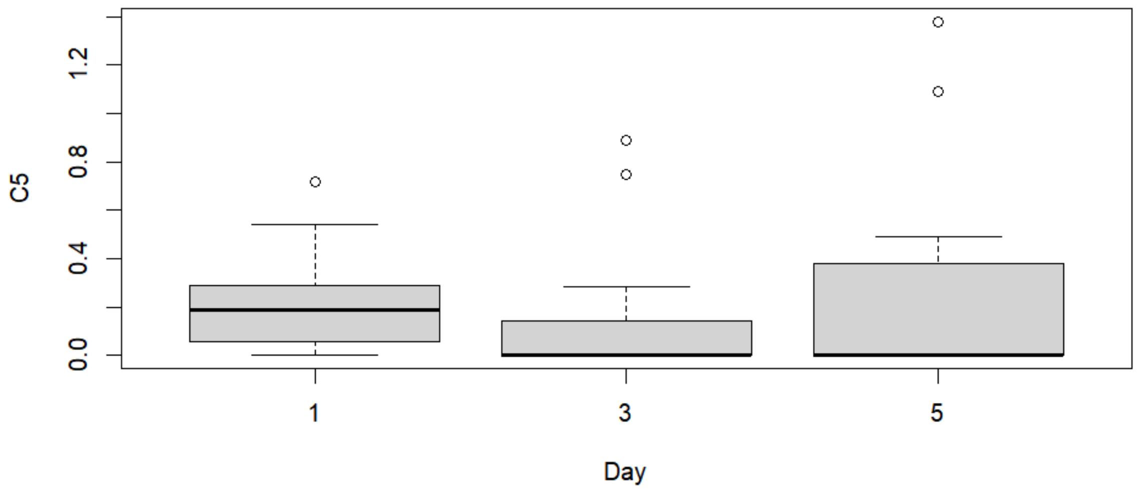


```
> aov(Control~Day)
Call:
   aov(formula = Control ~ Day)

Terms:
                     Day Residuals
Sum of Squares  1808.166  6412.784
Deg. of Freedom        2        30

Residual standard error: 14.62052
Estimated effects may be unbalanced

> aov(C1~Day)
Call:
   aov(formula = C1 ~ Day)

Terms:
                     Day Residuals
Sum of Squares  1491.129  3130.605
Deg. of Freedom        2        30
```

```
Residual standard error: 10.21536
Estimated effects may be unbalanced

> aov(C2~Day)
Call:
   aov(formula = C2 ~ Day)

Terms:
                     Day Residuals
Sum of Squares   424.019  4000.873
Deg. of Freedom        2        30

Residual standard error: 11.54827
Estimated effects may be unbalanced

> aov(C3~Day)
Call:
   aov(formula = C3 ~ Day)

Terms:
                      Day Residuals
Sum of Squares    89.0875 1466.8404
Deg. of Freedom         2        30

Residual standard error: 6.992473
Estimated effects may be unbalanced

> aov(C4~Day)
Call:
   aov(formula = C4 ~ Day)

Terms:
                      Day Residuals
Sum of Squares    4.14761 178.83057
Deg. of Freedom         2        30

Residual standard error: 2.44152
Estimated effects may be unbalanced

> aov(C5~Day)
Call:
   aov(formula = C5 ~ Day)

Terms:
                     Day Residuals
Sum of Squares  0.102055  4.000958
Deg. of Freedom        2        30

Residual standard error: 0.3651921
Estimated effects may be unbalanced

> AOV_CONTROL_NL <- aov(Control~Day)
> AOV_C1_NL <- aov(C1~Day)
> AOV_C2_NL <- aov(C2~Day)
> AOV_C3_NL <- aov(C3~Day)
> AOV_C4_NL <- aov(C4~Day)
> AOV_C5_NL <- aov(C5~Day)

> summary(AOV_CONTROL_NL)
            Df Sum Sq Mean Sq F value Pr(>F)
Day          2   1808   904.1   4.229 0.0241 *
Residuals   30   6413   213.8
---
Signif. codes:  0 ‘***’ 0.001 ‘**’ 0.01 ‘*’ 0.05 ‘.’ 0.1 ‘ ’ 1
> summary(AOV_C1_NL)
            Df Sum Sq Mean Sq F value Pr(>F)
Day          2   1491   745.6   7.145 0.0029 **
Residuals   30   3131   104.4
---
```

```
Signif. codes:  0 ‘***’ 0.001 ‘**’ 0.01 ‘*’ 0.05 ‘.’ 0.1 ‘ ’ 1
> summary(AOV_C2_NL)
            Df Sum Sq Mean Sq F value Pr(>F)
Day          2    424   212.0    1.59  0.221
Residuals   30   4001   133.4
> summary(AOV_C3_NL)
            Df Sum Sq Mean Sq F value Pr(>F)
Day          2   89.1   44.54   0.911  0.413
Residuals   30 1466.8   48.89
> summary(AOV_C4_NL)
            Df Sum Sq Mean Sq F value Pr(>F)
Day          2   4.15   2.074   0.348  0.709
Residuals   30 178.83   5.961
> summary(AOV_C5_NL)
            Df Sum Sq Mean Sq F value Pr(>F)
Day          2  0.102 0.05103   0.383  0.685
Residuals   30  4.001 0.13337
```

MONTECARLO ANALYSIS

```
# Paso 1: Crear un marco de datos
set.seed(23)
#Simulación de Monte Carlo Adhesion Energy Axon 48 H
v1<-runif(50,0,  2227.541)
v2<-runif(50,0,  111.876)
v3<-runif(50,0,  1341930.588)
v4<-runif(50,0,  20812.306)
datos <- data.frame(
  Treatments = rep(c("0.0um", "0.5um", "1.0um", "2.5um"), each = 50),
  Valor = c(v1,v2,v3,v4
  )
)
# Paso adicional: Calcular el valor absoluto de cada dato
datos$ValorAbsoluto <- abs(datos$Valor)
# Paso adicional: Expresar los valores absolutos como porcentajes del valor máximo
max_valor_absoluto <- max(datos$ValorAbsoluto)
datos$PorcentajeAbsoluto <- (datos$ValorAbsoluto / max_valor_absoluto) * 100
# Paso Adicional: Ordenar los niveles del factor para los tratamientos en el orden deseado
datos$Treatments <- factor(datos$Treatments, levels = c("0.0um", "0.5um", "1.0um", "2.5um"))
#Paso 2: Visualizar los valores absolutos para cada grupo en un boxplot
# Cargar la biblioteca ggplot2 si aún no está cargada
if (!requireNamespace("ggplot2", quietly = TRUE)) {
  install.packages("ggplot2")
}
library(ggplot2)
# Cargar la biblioteca RColorBrewer para utilizar la paleta "Set2"
if (!requireNamespace("RColorBrewer", quietly = TRUE)) {
```

```
  install.packages("RColorBrewer")
}
library(RColorBrewer)
# Cambiar automáticamente el color por grupos y agregar cuadrícula
bp <- ggplot(datos, aes(x = Treatments, y = PorcentajeAbsoluto, fill = Treatments)) +
  geom_boxplot() +
  labs(title = "Adhesion Energy 48 H", x = "Treatments µm", y = "Percentage of the Control") +
  scale_fill_brewer(palette = "Set3") +  # Paleta de colores "Set3"
  theme_classic() +
  theme(
    plot.title = element_text(hjust = 0.5, face = "bold"),
    axis.text.x = element_text(face = "bold"),
    axis.title.x = element_text(face = "bold"),
    axis.text.y = element_text(face = "bold"),
    axis.title.y = element_text(face = "bold"),
    panel.grid.major = element_line(color = "gray", linetype = "dashed")
  ) #+
  #scale_y_continuous(limits = c(30, 150)) # Establecer la escala del eje y de 30 a 150
# Mostrar el gráfico
print(bp)
# Paso 3: ANOVA
ajuste_anova <- aov(Valor ~ Treatments, data = datos)
summary(ajuste_anova)
# Paso 4: Dunnett's Test
library(DescTools)
# Realizar el test de Dunnett con "TN" como grupo de control
comparaciones_dunnett <- DunnettTest(x = datos$Valor, g = datos$Treatments, control = "0.0um")
print(comparaciones_dunnett)
```

RESULTADOS

NUCELEUS 24H

#Modulo Nucleus 24 H

v1<-runif(50,0, 2227.541)

v2<-runif(50,0, 111.876)

v3<-runif(50,0, 1341930.588)

v4<-runif(50,0, 20812.306)

diff   lwr.ci   upr.ci   pval

0.5um-0.0um -1199.344 -88021.66 85622.97 1.0000

1.0um-0.0um 636304.777 549482.46 723127.09 <2e-16 ***

2.5um-0.0um 11063.546 -75758.77 97885.86 0.9815

#Stiffness Nucleus 24 H

v1<-runif(50,0,1772.774)

v2<-runif(50,0, 7925.025)

v3<-runif(50,0, 7624.378)

v4<-runif(50,0, 41657.809)

$`0.0um`

diff   lwr.ci   upr.ci   pval

0.5um-0.0um 3104.382   67.37959 6141.384 0.0437 *

1.0um-0.0um 2702.381 -334.62078 5739.383 0.0930 .

2.5um-0.0um 18285.694 15248.69171 21322.696 <2e-16 ***

#Disipation Nucleus 24 H

v1<-runif(50,0, 25,674)

v2<-runif(50,0, 1,883)

v3<-runif(50,0, 8,945)

v4<-runif(50,0, 30,932)

diff   lwr.ci   upr.ci   pval

```
0.5um-0.0um -13.522908 -16.284563 -10.761253 < 2e-16 ***
1.0um-0.0um -10.226021 -12.987676  -7.464366 1.4e-15 ***
2.5um-0.0um   3.833843   1.072188   6.595498  0.0036 **
```

#Z Nucleus 24 H

```
v1<-runif(50,-162.142,   393.808)
v2<-runif(50,-118.006,   216.196)
v3<-runif(50,-124.845,   73.933)
v4<-runif(50,-209.33,     665.551)
               diff    lwr.ci     upr.ci    pval
0.5um-0.0um -100.1945 -175.0536  -25.33546  0.0050 **
1.0um-0.0um -181.7252 -256.5843 -106.86617 7.3e-08 ***
2.5um-0.0um  157.2206   82.3615  232.07964 6.0e-06 ***
```

#Deformation Nucleus 24 H

```
v1<-runif(50,0,  7.86)
v2<-runif(50,0,  20.133)
v3<-runif(50,0,  11.074)
v4<-runif(50,0,  7.197)
                diff     lwr.ci   upr.ci    pval
0.5um-0.0um  5.7571555  3.9812030 7.533108 9.8e-12 ***
1.0um-0.0um  0.8296069 -0.9463456 2.605559  0.5513
2.5um-0.0um -0.1715815 -1.9475339 1.604371  0.9917
```

#Adhesion Force Nucleus 24 H

```
v1<-runif(50,0,  133.605)
v2<-runif(50,0,  56.307)
v3<-runif(50,0.378,        127.736)
v4<-runif(50,0,  129.927)
```

```
                diff    lwr.ci     upr.ci   pval
0.5um-0.0um -46.835158 -62.34152 -31.328795  2e-10 ***
1.0um-0.0um -14.444248 -29.95061   1.062115 0.0741 .
2.5um-0.0um   1.577247 -13.92912  17.083610 0.9904
```

#Adhesion Energy Nucleus 24 H

```
v1<-runif(50,0,  10.475)
v2<-runif(50,0,  1.880)
v3<-runif(50,0,  8.971)
v4<-runif(50,0, 10.036)
                 diff    lwr.ci     upr.ci   pval
0.5um-0.0um -4.95472479 -6.094822 -3.8146280 <2e-16 ***
1.0um-0.0um -1.64396722 -2.784064 -0.5038704 0.0022 **
2.5um-0.0um  0.03449477 -1.105602  1.1745915 0.9997
```

AXON 24 H

#Modulo Axon 24 H

```
v1<-runif(50,0,4355.980)
v2<-runif(50,0,  3110.809)
v3<-runif(50,0,  12303.900)
v4<-runif(50,0, 9782.487)
            diff    lwr.ci    upr.ci    pval
0.5um-0.0um -881.7317 -1978.899  215.4358  0.1448
1.0um-0.0um 3389.6239  2292.456 4486.7915 8.6e-12 ***
2.5um-0.0um 3334.5386  2237.371 4431.7061 5.4e-11 ***
```

#Stiffness Axon 24 H

```
v1<-runif(50,0,671.324)
```

```
v2<-runif(50,0,  255.115)
v3<-runif(50,0,  72645.812)
v4<-runif(50,0, 52995.406)
                diff    lwr.ci    upr.ci   pval
0.5um-0.0um  -249.4065 -6153.784  5654.971 0.9993
1.0um-0.0um 34136.0257 28231.648 40040.403 <2e-16 ***
2.5um-0.0um 30991.2578 25086.880 36895.636 <2e-16 ***

#Disipation Axon 24 H
v1<-runif(50,0, 29.859)
v2<-runif(50,0,  6.967)
v3<-runif(50,0,  8.403)
v4<-runif(50,0, 30.288)
            diff     lwr.ci     upr.ci   pval
0.5um-0.0um -13.309645 -16.254937 -10.364353 <2e-16 ***
1.0um-0.0um -12.843171 -15.788464  -9.897879 <2e-16 ***
2.5um-0.0um   1.092995  -1.852297   4.038288 0.7098


#Z Axon 24 H
v1<-runif(50,-108.725,   508.9)
v2<-runif(50,-111.372,   154.513)
v3<-runif(50,-176.177,   94.135)
v4<-runif(50,-59.647,     530.289)
             diff     lwr.ci    upr.ci    pval
0.5um-0.0um -216.32573 -279.18394 -153.4675 5.1e-13 ***
1.0um-0.0um -287.26234 -350.12055 -224.4041 < 2e-16 ***
2.5um-0.0um   50.04357  -12.81464  112.9018  0.1503
```

```
#Deformation Axon 24 H
v1<-runif(50,0,  4.744)
v2<-runif(50,0,15.021)
v3<-runif(50,0,  10.743)
v4<-runif(50,0,  5.666)
  diff     lwr.ci   upr.ci    pval
0.5um-0.0um 4.9269849  3.5430816 6.310888   8e-14 ***
1.0um-0.0um 2.4292507  1.0453473 3.813154 0.00013 ***
2.5um-0.0um 0.6790722 -0.7048312 2.062976 0.51312

#Adhesion Force Axon 24 H
v1<-runif(50,0,  130.223)
v2<-runif(50,0.332,        114.687)
v3<-runif(50,1.243,        124.960)
v4<-runif(50,0,  127.173)
                diff    lwr.ci    upr.ci   pval
0.5um-0.0um -15.219411 -31.99851  1.559689 0.0847 .
1.0um-0.0um -13.402229 -30.18133  3.376872 0.1483
2.5um-0.0um   1.853945 -14.92516 18.633046 0.9877

#Adhesion Energy Axon 24 H
v1<-runif(50,0,  10.466)
v2<-runif(50,0.001,        6.995)
v3<-runif(50,0.009,        8.391)
v4<-runif(50,0, 9.793)

                diff    lwr.ci    upr.ci    pval
0.5um-0.0um -2.3605633 -3.574205 -1.146921 2.6e-05 ***
```

1.0um-0.0um -1.9097308 -3.123373 -0.696089 0.00072 ***

2.5um-0.0um -0.1042706 -1.317912 1.109371 0.99412

NUCLEO 48 H

```
#Modulo Nucleus48 H
v1<-runif(50,0,102866.354)
v2<-runif(50,0,  370598.199)
v3<-runif(50,0,  234837.269)
v4<-runif(50,0, 8546.546)
               diff    lwr.ci    upr.ci    pval
0.5um-0.0um 129552.43  97926.24 161178.61 < 2e-16 ***
1.0um-0.0um  53573.38  21947.20  85199.57 0.00024 ***
2.5um-0.0um -52940.48 -84566.67 -21314.29 0.00036 ***

#Stiffness Nucleus48 H
v1<-runif(50,0, 11759.372)
v2<-runif(50,0,  1710.795)
v3<-runif(50,0,  42270.922)
v4<-runif(50,0, 25635.123)
               diff    lwr.ci    upr.ci    pval
0.5um-0.0um -5764.527 -9095.438 -2433.617 0.00018 ***
1.0um-0.0um 13452.890 10121.980 16783.801 < 2e-16 ***
2.5um-0.0um  8543.971  5213.061 11874.882   3e-08 ***

#Disipation Nucleus48 H
v1<-runif(50,0, 31.961)
v2<-runif(50,0,  32.701)
v3<-runif(50,0,  30.965)
```

v4<-runif(50,0, 26.435)

```
              diff    lwr.ci    upr.ci   pval
0.5um-0.0um -1.471390 -5.635711 2.6929305 0.7383
1.0um-0.0um -3.308982 -7.473303 0.8553386 0.1513
2.5um-0.0um -2.372903 -6.537224 1.7914178 0.3930
```

#Z Nucleus48 H

v1<-runif(50,-102.851,   521.339)

v2<-runif(50,-105.832,   613.62)

v3<-runif(50,-310.482,   508.62)

v4<-runif(50,-211.768,   405.235)

```
                diff     lwr.ci     upr.ci    pval
0.5um-0.0um    9.179352  -85.62513 103.9838340  0.9917
1.0um-0.0um -170.408886 -265.21337 -75.6044039 8.9e-05 ***
2.5um-0.0um  -95.631119 -190.43560  -0.8266367  0.0476 *
```

#Deformation Nucleus48 H

v1<-runif(50,0, 4.797)

v2<-runif(50,0, 3.767)

v3<-runif(50,0, 46.564)

v4<-runif(50,0, 13.524)

```
               diff     lwr.ci    upr.ci   pval
0.5um-0.0um -0.3458803 -3.7461647  3.054404 0.9904
1.0um-0.0um 19.2816111 15.8813267 22.681895 <2e-16 ***
2.5um-0.0um  3.9918265  0.5915421  7.392111 0.0165 *
```

#Adhesion Force Nucleus48 H

v1<-runif(50,0, 134.792)

```
v2<-runif(50,0,  134.705)
v3<-runif(50,0,  134.494)
v4<-runif(50,0,  120.554)

                diff    lwr.ci    upr.ci   pval
0.5um-0.0um  -7.828862 -25.65423  9.996510 0.5973
1.0um-0.0um -12.101154 -29.92653  5.724218 0.2561
2.5um-0.0um  -4.640218 -22.46559 13.185154 0.8714
```

```
#Adhesion Energy Nucleus48 H
v1<-runif(50,0,  10.006)
v2<-runif(50,0, 9.888)
v3<-runif(50,0,  9.552)
v4<-runif(50,0, 9.051)

                diff    lwr.ci    upr.ci   pval
0.5um-0.0um -0.6376080 -1.946159 0.6709433 0.5186
1.0um-0.0um -1.1034922 -2.412043 0.2050590 0.1187
2.5um-0.0um -0.2841223 -1.592673 1.0244290 0.9192
```

AXON 48 H

```
#Modulo Axon 48 H
v1<-runif(50,0,11575.297)
v2<-runif(50,0,  13905.700)
v3<-runif(50,0,  640075.352)
v4<-runif(50,0, 37881.792)
```

```
            diff    lwr.ci    upr.ci   pval
0.5um-0.0um    510.8431 -40994.42  42016.11 1.0000
1.0um-0.0um 297577.8017 256072.54 339083.07 <2e-16 ***
2.5um-0.0um  15896.9765 -25608.29  57402.24 0.6904

#Stiffness Axon 48 H
v1<-runif(50,0,671.324)
v2<-runif(50,0,  255.115)
v3<-runif(50,0,  72645.812)
v4<-runif(50,0, 52995.406)
            diff    lwr.ci    upr.ci   pval
0.5um-0.0um  -249.4065 -6153.784  5654.971 0.9993
1.0um-0.0um 34136.0257 28231.648 40040.403 <2e-16 ***
2.5um-0.0um 30991.2578 25086.880 36895.636 <2e-16 ***

#Disipation Axon 48 H
v1<-runif(50,0, 32.353)
v2<-runif(50,0, 9.525)
v3<-runif(50,0,  32.55)
v4<-runif(50,0, 10.056)
            diff     lwr.ci      upr.ci    pval
0.5um-0.0um -13.42130 -16.585197 -10.2573938 < 2e-16 ***
1.0um-0.0um  -2.77696  -5.940862   0.3869415  0.0993 .
2.5um-0.0um -12.28921 -15.453111  -9.1253080 2.2e-16 ***

#Z Axon 48 H
v1<-runif(50,-59.252,     562.52)
v2<-runif(50,-50.736,     152.333)
v3<-runif(50,-71.234,     572.373)
```

```
v4<-runif(50,-99.151,     83.09)
                diff    lwr.ci      upr.ci    pval
0.5um-0.0um -239.29079 -301.0559 -177.525635 7.8e-16 ***
1.0um-0.0um  -56.77546 -118.5406    4.989698  0.0793 .
2.5um-0.0um -282.60010 -344.3653 -220.834942 < 2e-16 ***

#Deformation Axon 48 H
v1<-runif(50,0,  6.624)
v2<-runif(50,0, 6.197)
v3<-runif(50,0,  8.915)
v4<-runif(50,0,  8.14)
                diff      lwr.ci    upr.ci   pval
0.5um-0.0um -0.5986609 -1.61373732 0.4164156 0.3652
1.0um-0.0um  0.5007485 -0.51432794 1.5158249 0.5089
2.5um-0.0um  1.0835100  0.06843355 2.0985864 0.0333 *

#Adhesion Force Axon 48 H
v1<-runif(50,0,  134.661)
v2<-runif(50,0,  134.613)
v3<-runif(50,0,  134.559)
v4<-runif(50,0,  115.507)
                diff    lwr.ci    upr.ci   pval
0.5um-0.0um  -7.801559 -25.46824  9.865121 0.5933
1.0um-0.0um -11.996410 -29.66309  5.670271 0.2560
2.5um-0.0um  -7.553846 -25.22053 10.112834 0.6166

#Adhesion Energy Axon 48 H
v1<-runif(50,0,  10.098)
v2<-runif(50,0, 9.511)
```

```
v3<-runif(50,0,  10.040)

v4<-runif(50,0, 8.081)
```

```
              diff    lwr.ci    upr.ci   pval
0.5um-0.0um -0.8802723 -2.163424 0.4028789 0.2484
1.0um-0.0um -0.9235126 -2.206664 0.3596386 0.2145
2.5um-0.0um -0.9101707 -2.193322 0.3729805 0.2247
```
